# Advancements in CRISPR-Based Biosensing for Next-Gen Point of Care Diagnostic Application

**DOI:** 10.3390/bios13020202

**Published:** 2023-01-29

**Authors:** Akash Kumaran, Nathan Jude Serpes, Tisha Gupta, Abija James, Avinash Sharma, Deepak Kumar, Rupak Nagraik, Vaneet Kumar, Sadanand Pandey

**Affiliations:** 1 Faculty of Applied Sciences and Biotechnology, Shoolini University, Solan 173229, Himachal Pradesh, India; 2 Department of Pharmaceutical Chemistry, School of Pharmaceutical Sciences, Shoolini University, Solan 173229, Himachal Pradesh, India; 3 Department of Natural Science, CT University, Ludhiana 142024, Punjab, India; 4 Department of Chemistry, College of Natural Sciences, Yeungnam University, Gyeongsan 38541, Gyeongbuk, Republic of Korea

**Keywords:** CRISPR, Cas effector proteins, biosensor, diseases, E-CRISPR

## Abstract

With the move of molecular tests from diagnostic labs to on-site testing becoming more common, there is a sudden rise in demand for nucleic acid-based diagnostic tools that are selective, sensitive, flexible to terrain changes, and cost-effective to assist in point-of-care systems for large-scale screening and to be used in remote locations in cases of outbreaks and pandemics. CRISPR-based biosensors comprise a promising new approach to nucleic acid detection, which uses Cas effector proteins (Cas9, Cas12, and Cas13) as extremely specialized identification components that may be used in conjunction with a variety of readout approaches (such as fluorescence, colorimetry, potentiometry, lateral flow assay, etc.) for onsite analysis. In this review, we cover some technical aspects of integrating the CRISPR Cas system with traditional biosensing readout methods and amplification technologies such as polymerase chain reaction (PCR), loop-mediated isothermal amplification (LAMP), and recombinase polymerase amplification (RPA) and continue to elaborate on the prospects of the developed biosensor in the detection of some major viral and bacterial diseases. Within the scope of this article, we also discuss the recent COVID pandemic and the numerous CRISPR biosensors that have undergone development since its advent. Finally, we discuss some challenges and future prospects of CRISPR Cas systems in point-of-care testing.

## 1. Introduction

Access to rapid and convenient diagnostic kits is a key element of any healthcare system. Point-of-care testing (POCT), also referred to as bedside or extra laboratory testing [1], can be described as “testing at or near the site of patient care whenever the medical care is needed” [2]. POC devices generally aim to follow the ASSURED criteria (affordable, sensitive, specific, user-friendly, rapid and robust, equipment-free, and deliverable to end-users) to provide rapid and selective patient information on-site to provide personalized care or rapidly provide pre-diagnose of an infection, often bypassing traditional time-consuming methods and the need for specialized laboratories [3]. Moreover, the recent pandemic caused by the rapid spread of the severe acute respiratory syndrome coronavirus2 (SARS-CoV-2) RNA virus has exposed the need for better, more dependable, and field-deployable POCT solutions, as the previously available testing methodologies, which included the use of immunoassays, PCR testing, etc., were too cumbersome and proved ineffective in detecting low viral population in patient samples during early stages of infection [4,5].

Many infectious pathogens have plagued humans, including acute infections such as hepatitis B, hepatitis C, *Helicobacter pylori*, dengue, HPV, and chlamydia, as well as food poisoning pathogens such as *E. coli*, *Salmonella*, *Staphylococcus aureus*, etc., Numerous infections such as hepatitis B and hepatitis C, HPV, and chlamydia can be transformed into chronic stages, thereby affecting a person’s lifespan. Diseases such as the human immunodeficiency virus (HIV), hepatitis virus (HBV and HCV), and tuberculosis are spread widely and are vicious killers, and these features describe the importance of these diseases for global health. Since viral infections are newly emerging and also re-emerging, recognizing a virus’s etiology is mandatory and vital to providing suitable treatment, thereby reducing the risk to patients and preventing the spread of the disease. Jennifer Doudna and Emmanuelle Charpentier’s work on CRISPR introduced next-generation-based genome editing and propelled the creation of new CRISPR-based diagnostics and the gene editing field and was awarded the 2020 Nobel prize in chemistry [6].

The modern development of Clustered Regularly Interspaced Short Palindromic Repeats (CRISPR) technology, in addition to revolutionizing the genome-editing field, has given rise to a new generation of molecular diagnostic and POCT techniques [7]. CRISPR-Cas utilizes the adaptive immune system that preys on foreign nucleic acids and is broadly found in bacteria and archaea. The CRISPR-Cas system is generally composed of Cas proteins and CRISPR RNA (crRNA) [8]. The CRISPR-Cas family system is divided into two types and subdivided into six distinctive types based on their protein usage. The Class 1 system has types I, III, and IV, while the Class 2 system has types II, V, and VI [9]. CRISPR-Cas technology also can adapt itself and bring in a change in other living entities, such as plants. To provide resistance against plant diseases, CRISPR-Cas has been modified to prevent the plants from being infected [10]. The CRISPR-Cas9-based genome editing methods are able to safeguard plants. Plants that showed resistance include *N. benthamiana*, and *Arabidopsis* showed resistance against *Merremia mosaic virus* (MeMV), *Beet severe curly top virus* (BSCTV), *Beet curly top virus* (BCTV), *Bean yellow dwarf virus* (BeYDV), and *Tomato yellow leaf curl virus* (TYLCV) [10].

**Figure 1:**
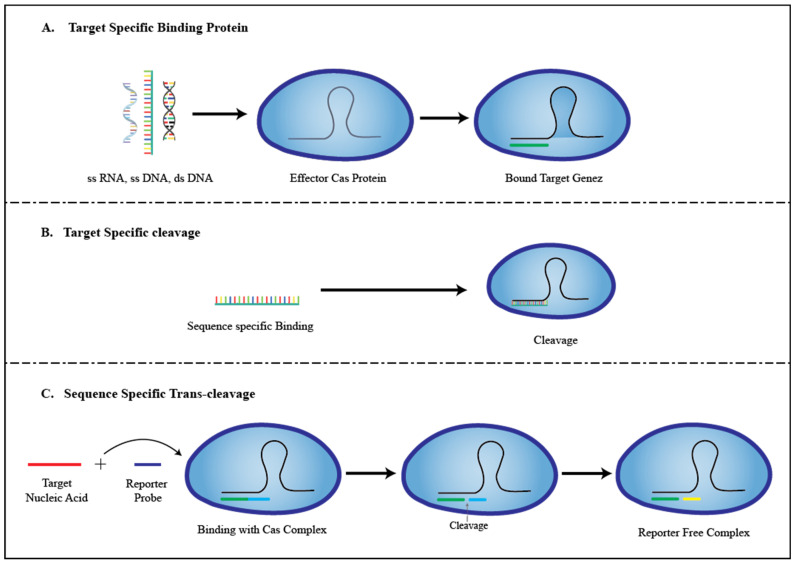
Step mechanism used by the CRISPR-Cas system to identify, isolate, and/or knockout a gene sequence. (**A**) Target-specific binding: The process of acquiring a spacer starts with Cas1 and Cas2 recognizing the invasive DNA and cleaving a protospacer. In order to replicate the direct repeat and repair the CRISPR, the protospacer is ligated to the direct repeat next to the leader sequence. The Cas protein that is catalytically inactive is bent to the target gene, which is complementary to gRNA. (**B**) Biogenesis and target-specific cleavage: The CRISPR sequence must be converted into CRISPR-RNA (crRNA), which later directs the Cas nuclease to the target during the interference stage. The crRNA is first produced as a single lengthy transcript that covers the majority of the CRISPR array. The Cas proteins then split this transcript into crRNAs. The way that different CRISPR-Cas systems create crRNAs varies. The target gene is cleaved by Cas proteins thatare followed by sequence-specific binding. (**C**) Sequence-specific trans cleavage: The Cas protein non-specifically cleaves the ssDNA or ssRNA upon binding to the target gene, thereby freeing the reporter probe.

In lieu of these advances, there are several CRISPR-based DNA or RNA detection technologies available, including DNA Endonuclease Targeted CRISPR Trans reporter (DETECTR) (using Cas12a, Cas14), One-Hour Low-cost Multipurpose highly Efficient System (HOLMES) (using Cas12a), Cas12b mediated DNA detection (CDetection) (using Cas12b), Electrochemical CRISPR (E-CRISPR) (using Cas12a), andSpecific High Sensitivity Enzymatic Reporter UnLOCKING (SHERLOCK) (using Cas13a), which have been developed by the use of CRISPR Cas’s target-induced trans-cleavage activity system as a signal amplifier [10,11]. Figure 1 depicts the general mechanism used by CRISPR-Cas sensors. Researchers have attempted to build POCT solutions using these CRISPR-based approaches for use in clinical settings to generate highly accurate and quick diagnostic results by rapidly detecting clinically relevant molecules such as nucleic acids (DNA, RNA) and small biological molecules with high specificity and sensitivity (down to attomolar [aM] concentration limits of detection (LOD)) [10,11], so that the proper therapy may be given, resulting in better pharmacoeconomic outcomes. CRISPR can also be used as a biosensing strategy to discriminate point mutations, which has significant importance in genetic disorder analysis [11].

**Figure 2:**
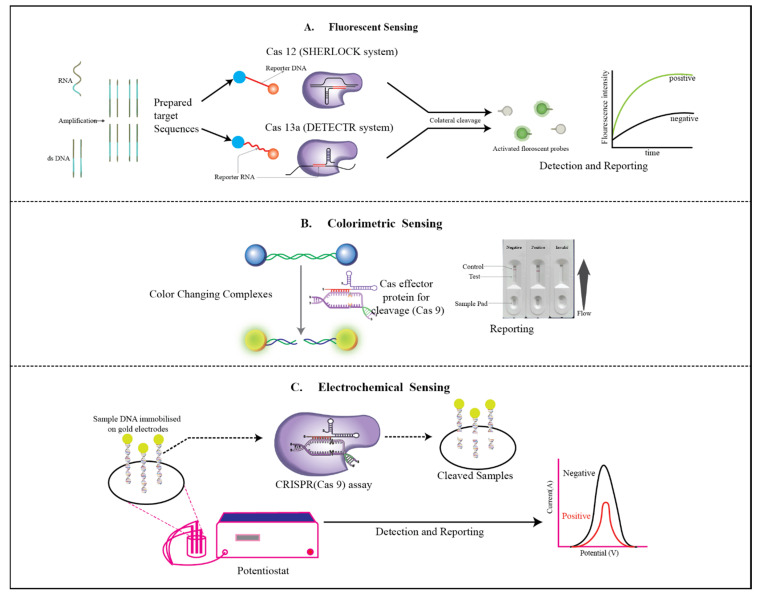
Three widely used signal detection techniques can be monitored to detect the existence of the target gene. (**A**) Fluorescence-based sensing uses target sequences prepared with fluorescent probes to detect target sequences based on the changes in fluorescence intensity. A higher intensity than the control denotes a positive result; examples are SHERLOCK and DETECTR. (**B**) Colorimetric sensing uses color-changing complexes attached to CRISPR-Cas as complexes to detect target sequences. When the complexes are cleaved using targeted the CRISPR assay (Cas9), a color change is observed. (**C**) Electrochemical sensing detects the target sequence based on changes in electrical potential using a potentiostat.

This review focuses on biosensing methods such as fluorescent, colorimetric, and electrochemical biosensors that integrate CRISPR-Cas systems in POC operations. In doing so, we also review various emerging CRISPR-based diagnostic platforms such as SHERLOCK, DETECTR, and E-CRISPR and we have mentioned their working mechanism that are shown in Figure 2. We have also mentioned their role in the detection of common viral and bacterial infections. We also discuss the recent outbreak of COVID-19 and its role in the advancement of CRISPR-based biosensors.

## 2. Benefits of CRISPR-Based Biosensors in Point-of-Care Systems

In environments where resources are lacking, point-of-care testing (POCT) necessitates instruments that work independently of the standard laboratory framework [12]. The goal of POCT is to offer clinicians real-time information on a patient’s state so that this information may be used to make better treatment decisions that enhance patient outcomes, such as lowering criticality, morbidity, and mortality. Point-of-care testing can be done in a variety of settings, including the hospital, at home, or elsewhere. Depending on the format, point-of-care equipment can be classified as “transportable”, “portable”, or “handheld” [12]. According to the World Health Organization’s (WHO)ASSURED criteria, newly developed POCT implementations must be affordable, highly sensitive, selective, user-friendly, quick and robust, independent of complex equipment, and deliverable to the end-user, i.e., the patient. Newly developed novel pathogen-recognition technologies that use modified nucleic acid binding and cleavage proteins may be able to help close this gap, as some of the systems already meet the ASSURED standards [13]. Due to recent interest in the integration of CRISPR Cas systems in diagnostic applications through nucleic acids, detection has the potential to decrease the medical field’s dependency on PCR-based instrumentation. Short detection cycles, low cost, great sensitivity, and the flexibility to be paired with numerous readout methods [14] are all aspects that make it suited for on-site POCT (point-of-care testing). The most current CRISPR-associated protein technologies (NASBACC, SHERLOCKv2, DETECTR, HOLMES) are purported to be affordable (less than USD 1 in certain cases) and straightforward to implement.

## 3. Role of CRISPR in the Development of Biosensors

A biosensor is a device that quantifies and evaluates chemical or biological reactions by initiating signals in comparison to the concentration of the analyte in a reaction. Biosensors currently are used in various applications such as the monitoring of disease, pollutant detection, drug discovery, the discovery of disease-causing microorganisms, and disease-indicating biomarkers. Every biosensor has an attribute or a characteristic it possesses, and its working performance is based on this, including selectivity, reproducibility, stability, sensitivity, and linearity [15].

In this modern world, the need for rapid, cost-effective, early detecting, and accurate biosensors in many fields has pushed for the development of biosensors to a greater extent, owing to their prompt signal readouts, economical transduction elements, and manageable sensing platforms [16]. Therefore, biosensing is elementary for diagnostic testing that comprises lab-based medical diagnostics and non-laboratory-based POCT, which is critical for handling the well-being of living organisms who reside in restricted areas with limited resources and a high probability of spreading infectious disease [17]. Biosensing technologies and current and future devices will play dominant roles in shaping the future of innovation [18]. Generally, characteristics of a biosensor include its reliability, accuracy, sensitivity, screening of the samples, and presentation of results in a short time-period [19]. Various biosensors have successfully been developed and are in development with regards to their characteristics, but each biosensing technology has its limitations and challenges, which include low selectivity and specificity against targets, and low flexibility [20]. Detection sensitivity can be improved by finer sample preparation, but that also increases its total assay time [20]. While all these problems hinder the potential of biosensor technology, currently various innovative technologies have emerged to advance biosensor technology to a greater extent in various areas.

The innovation of CRISPR has revamped the understanding of biosensing systems and has also allowed the CRISPR system to be used in various applications, for instance, genomic editing, biomarkers, transcription regulations, and much more [21]. CRISPR is a prokaryotic adaptive-based immune system, i.e., available in archaea and bacteria; the CRISPR-Cas system has seen rapid growth and advancement in precise RNA-navigated genome editing in various molecular systems [22].

The CRISPR-Cas protein family includes the programmable endonuclease enzymes Cas9, dCas9, Cas13a, Cas12a, and Cas14, and many more that are very specific and selective when led by a particular gRNA complementary to the recognition site on the desired DNA/RNA [22]. The CRISPR biosensor system is extremely sensitive and specific with fast, simple, and strong performance, as it possesses the unique properties of sequence-specific DNA targeting and cleavage that make CRISPR-Cas technology a novel technology for genomic and molecular editing [23]. The usage of CRISPR technology has already been applied in the area of research and treatment of diseases, including cancer, AIDS, CNS diseases, and monogenic diseases [24]. The CRISPR-Cas families of Cas9, Cas12, Cas13, and Cas14 have already been developed and also are in development in the context of biosensing technology to provide precise detection and diagnostics [25]. Due to their cleavage advantages, CRISPR-based biosensors have been developed for RNA and DNA assays, Cas12a-based DETECTR, and SHERLOCK, an architect in CRISPR-established biosensors to detect nucleic acid [26]. It is also been reported that SHERLOCK can be implemented to monitor the coronavirus (SARS-CoV-2) in clinical samples owing to its superiority over RT-PCR [27]. CRISPR-Cas technology is being continuously optimized with better performance and with innovative ideas for its rapid growth in various fields [28]. The detection of disorders based on nucleic acids can be achieved using the CRISPR-Cas system, and various detection platforms have been implemented for this, such as E-CRISPR, voltammetry, fluorescence, and colorimetry [29].

### 3.1. E-CRISPR

The electrochemical-based biosensor technique has earned a lot of attention due to its simple, cost-effective, easy way to construct; its rapid response to detection; its high sensitivity high selectivity; and its small size [30]. An electrochemical biosensor is an analytical device that can recognize a particular biological molecule in an electrode and convert its biological contents into a measurable output signal by transduction (electronic) detection of small or nucleic molecules, which is the most significant advantage of electrochemical biosensors; these sensors also need a well-positioned surface area so as not to disturb the electron flow rate [31]. Electrochemical biosensors also have the capability of being manufactured into a portable kit to be used for various monitoring purposes [32]. Due to their properties, the advantages of utilizing electrochemical biosensors for various diagnostics applications, research areas, and also in POCT are immense. A crucial challenge faced by this sensor system is its accuracy, but researchers have come up with a brilliant solution by combining CRISPR-Cas technology with this biosensor [33], and a lot of CRISPR-based biosensors have successfully been identified. In this review, we look into E-CRISPR, which is based on CRISPR-Cas12a.

A pioneered work in inventing an aptamer-CRISPR-based biosensor is the CRISPR-Cas12a-based biosensor, otherwise known as E-CRISPR [34,35], by taking advantage of the trans-acting cleavage properties of single-strand DNA (ssDNA) reporters, initiated through the cis-acting excision of the targeted DNA by the CRISPR-Cas system of Csm6, Cas12a, and Cas13. Cas-crRNA has been used for detection purposes by using the colorimetric system and the fluorescent transduction system [35,36], and Dai et al. created a new biosensing system using the properties of CRISPR-Cas12a.

E-CRISPR is a basic endonuclease transduction technique that is applied for sensing systems based on the nuclease and facilitates the detection of various analyte categories, as it is been used for CRISPR types III, V, and VI. For developing the electrochemical biosensor, Ag/Agcl was well-suited to be the reference electrode, and also a replaceable three-electrode sensor with micro-fabricated gold was used, and for the counter and the working electrode, gold was deployed. The working of the Cas12a-crRNA duplex is that the Cas protein specifically perceives and cleaves a single strand of nucleic acid that has been established on the PAM sequence (protospacer adjacent motif) of the target and interdependent in the middle of the target and crRNA. A specific nucleic acid sequence is situated in the opposite strand to the recognition strand, and the PAM recognition relies on this specific nucleic acid strand, and the CRISPR-Cas protein acts as a DNA helicase; with the identification of the PAM sequence by CRISPR protein, it unwinds its target DNA [36]. Due to the ongoing activity, the target strand becomesseparated, and the cleavage activity that interrelates crRNA and the target is activated [36]. To acquire electrochemical transduction of the CRISPR detection signal, the trans-cleavage activity (the cis-cleavage commenced trans-cleavage activity of effect Cas12a) therefore cleaves the nonspecific single-strand DNA (ssDNA) by the electrochemical method, and it is fascinating to take note as the trans-cleavage does not act concurrently with cis-cleavage. An ssDNA reporter that is nonspecific is designed with methylene blue (MB), which is used as an electrochemical tag for signal transduction; the ssDNA was purposefully designed to be incorporated with a gold electrode of the electrochemical cell, and a thiol moiety was conjointly designed for bonding directly to the sensor surface in favor of acquiring the signal electrically. Therefore, the transfer process of electrons between the redox-active particles present on the ssDNA and the gold electrode can be electrochemically instigated and then transduced. Thus, due to the existence of the target, the working of the Cas12a trans-cleavage activity is triggered; it cleaves the unified methylene blue (MB) reporter ssDNA from the surface of the electrode, promoting the reduction of methylene blue signal transduction and contrast in the absence of the target. The trans cleavage activity of the Cas12a protein is suppressed, wherein it retains the MB-ssDNA reporter directly on the surface. Due to its simple design and effective cost-cutting CRISPR transduction plan, the usage of the MB-ssDNA electrode applies to any CRISPR (type-III, V, VI) system [36,37].

With its great ability, E-CRISPR also comes with a few challenges, as it utilizes ssDNA to transduce on the electrode for sensing purposes. Two possible problems thus arise:The first one is that due to the long distance between the reporter ssDNA and electrodes, the electron transfer rate is affected, therefore resulting in a lower electrochemical response. The second is having low cleavage efficiency due to the steric-hindrance effect, which influences the interaction between the Cas12a protein and the ssDNA reporter [37].

To solve the hindering problem, the design of the ssDNA, which was linear, was changed to the hairpin type, where both ends are connected to the methylene blue and electrode, respectively, and this tweaking has drastically improved the overall analytical performance of the electrochemical biosensor (hpDNA). Another electrochemical biosensor known as E-DNA (electrochemical DNA) has been advanced, wherein the gold electrode is attached to the electrochemical cell by methylene blue-ssDNA [37].

To determine its feasibility and generality of detection, E-CRISPR was used to test nucleic acids of viruses such as human papillomavirus (HPV-16) and parvovirus B19 (PB-19) [38]. There is great potential for this analysis, as an LOD (experimental limit of detection) of 50 pM (picomolar) was obtained, and this LOD surpassed the previously published LOD for nucleic-acid detection [38].

E-CRISPR was also experimented with as a detection system for proteins by taking advantage of its detection capability; therefore, to detect protein, an aptamer ssDNA was utilized as a recognition element [39], and transforming growth factor (TGF-b1) was used as an experimental protein, and it was found that the aptamer complex can stop Cas12a from cleaving the reporter ssDNA [40] due to its wide range spectrum and portability it must use for POC diagnosis. It is also worth noting that for protein detection using E-CRISPR, increasing the trans-cleavage activity leadsto an upper detection resolution, and this strategy can be used to strengthen the detection performance, as it can be employed to tune the detection limit and dynamic range of E-CRISPR [40].

The inception of electrochemical biosensors in areas of POCT diagnosis has tackled issues such as the usage of operators, high cost, and expensive diagnostic treatment [41], and with the integration of CRISPR-based electrochemical biosensors, simple, portable kits have been made, and E-CRISPR has exhibited a cost-effective, sensitive, accurate, cost-effective, and commercialized platform for POCT diagnostics systems.

### 3.2. Colorimetry

Colorimetry is the science of quantification of color, substituting subjective responses of colors with an objective numerical system. The colorimeter is a device that is generally used to measure the color of the test sample and is the best alternative for quality detection [42]. Colorimetric biosensing approaches are extensively used for recognizing targets by the naked eye and are easily movable. These qualities enabled us to evolve an uncomplicated colorimetric contagion discovery system using the CRISPR-Cas system. The CRISPR-linked Cas system has progressed into enlightened gene-editing techniques; therefore, Moon et al. devised a mechanism in which RNA is present in the viral lysate that is identified by the dCas9/gRNA complex, which can stick to RNA in a programmed way accompanied by biotin-PAMmer [43]. PAM is a 2–6-base pair DNA sequence immediately succeeding the DNA sequence thatis targeted by the Cas9 nuclease-carrying oligonucleotide called PAMmer. As the Cas9 target pursuit method relies on the PAM sequence, applying the CRISPR/dCas9 system, the target RNA can be entirely recognized by the existence of PAMmers [43]. The immobilized well microplates attached to dCas9/gRNA were enriched with Biotin-PAMmer, and, additionally, the biotin was attached to the 3′-ends of the PAMmer sequence, favoring the tie-up of streptavidin-HRP. The streptavidin-horseradish peroxidase (HRP)/′,′-tetramethylbenzidine (TMB) response permitted the colorimetric finding of the contagion with the help of the naked eye or by the easy dimension of OD. By following this method, we can also detect the SARS-CoV-2, pH1N1, and oseltamivir-resistant pH1N1/H275Y viruses [43].

Colorimetric detection is superior for point-of-care applications as, without the requirement of an excitation light source or sophisticated equipment, it generates a visible signal [44]. Because of surface plasmon resonance (SPR), unique distance-dependent optical signals were generated by the aggregation and dispersion behavior of AuNPs. Hence, the viewed absorption peak is relocated to shorter or longer wavelengths, and contemporaneously the tincture of the colloidal solution is altered between red and purple. The trans cleavage of ssDNA substrates as the linkers of AuNPs, which control the aggregation and dispersion behavior, were engaged by the initial colorimetric assay conjugated with CRISPR-Cas12a, and binary color readouts were exposed by a sequence-specific plasmonic sensing platform to minimize the impact of environmental factors that gave rise to false assets or liabilities, due to orthogonality between the binary color channels [45].

Moreover, colorimetric code platforms were elaborated depending on programmable CRISPR technology for the detection of telomerase acts. The discovery is characterized by three distinct colorimetric codes, i.e., positive code (P), negative code (N), and false-negative code (FN), for the identification of the false-negative results arising from the PCR inhibitor, permitting speedy readout of telomerase act analysis and to make the judgment of the analysis accessible and user-friendly to a greater extent [46]. Despite this, enhanced colorimetry known as the magnetic pull-down-assisted colorimetric detection method was developed based on CRISPR/Cas12a (M-CDC), consequently assuring improved detection sensitivity and specificity [47].

### 3.3. Fluorescent Sensor

The detection of nucleic acids using fluorescence readout methods plays a vital role in the detection of tumors and viral and bacterial infections in the clinical setting, as it provides a user-friendly and quick method of reading sample results. Concerning CRISPR-based nucleic acid detection when a single-stranded DNA fluorescent probe is injected into a sample system, as a part of the CRISPR Cas protein complex, and when the target nucleic acids are present, the fluorescent probe will be cut to create a fluorescence signal [48]. At times, nucleic acid amplification strategies such as circle amplification (RCA), polymerase chain reaction (PCR), enhanced strand displacement amplification (E-SDA), recombinase polymerase amplification (RPA), loop-mediated isothermal amplification (LAMP), and rolling or catalytic hairpin assembly (CHA) are used to preprocess the sample to increase the concentration of nucleic acid to enable the detection of nucleic acids present in minuscule quantities [48].

Fluorescent-based nucleic acid detection that employs CRISPR systems has presently been employed in platforms, namely, SHERLOCK and DETECTR. Zhang’s lab adapted the natural RNase action of the Cas13 protein complex, which enabled the SHERLOCK system to be developed [48], while the non-specific ssDNA degradation of Cas12awas used by Doudna’s lab to develop the DETECTR platform [49].

The cleavage and degradative activity of Cas13 and Cas12a on the region surrounding the target ssRNA and ssDNA enable the detection of a free fluorescent reporter by SHERLOCK and DETECTR platforms [50]. This reporter’s measurable signal may be monitored and quantified to detect the existence and amount of DNA, RNA, or a gene of interest. SHERLOCK and DETECTR work together to highlight the capabilities of CRISPR-based diagnostics. dsDNA cleavage is an intrinsic property of Cas12a and Cas9 variants of the CRISPR Cas family of proteins. Cas12a recognizes a distinct PAM (protospacer adjacent motif) site on the target gene and creates 5′ and 3′ staggered ends after it splices the dsDNA [50]. One benefit of the Cas12a protein, as an enzyme, is that despite its small amplicon concentration, it can quickly enhance the fluorescence signal [50].

The DETECTR system uses the ssDNA targeting mechanism of Cas12a to target a specified DNA sequence via a cRNA [50]. If the Cas12a protein response to pathogenic DNA is supplemented with single-stranded DNA reporters (probes) and subsequently combined with the organic samples, there action of the crRNA-dependent detection of pathogenic nucleic acids by the Cas12a complex triggers ancillary activity that destroys the DNA probes [51]. The construction of these DNA probe reporters is such that they are connected to a fluorophore on one end, and the other end is attached to a quencher. Fluorophores are released during the degradation of the DNA probes, resulting in steady and strong fluorescent signals that can be measured by a fluorimeter. DETECTR has also been paired with an isothermal preamplification phase to increase the concentration oftarget sequences (RPA) [51]. As a result, DETECTR enables the rapid and accurate identification of nucleic acids in a diverse sample for a wide range of molecular and medical diagnostic purposes.

Cas13 could be programmed to target a relevant ssRNA such as a virus or pathogen-specific sequence using crRNA. The Cas13 complex cancleave the neighboring ssRNA molecules promiscuously after it detects and attaches to the encoded sequence. A quenched fluorescent ssRNA reporter is introduced to the process in SHERLOCK (Sensitivity Enzymatic Reporter UnLOCKING). The “activated” Cas13 cleavage of the quenchable fluorescent RNA gives out a measurable signal that signals the presence of the targeted ssRNA [52].

SHERLOCK, as established by Zhang’s lab in 2017, could consistently discriminate between Zika and a similarly related virus, Dengue, from diverse sample sources. SHERLOCK was also capable of detecting low-frequency cancer mutations in cell-free DNA fragments, as well as health-related single nucleotide polymorphisms (SNPs) in human saliva [52].

## 4. CRISPR-Based Biosensor in Diagnosis (Diseases)

In recent decades the rise of infectious pathogens is a worrying threat to the lives of Mankind over the past decades and the emergence of various pathogens is way faster than any other thing and due to huge populations they can easily spread over vast areas and even globally quickly to cause a pandemic [53] and there are a lot of examples to name, that includes Zika virus, Ebola virus, Influenza A (pH1N1) [54], the Middle East respiratory syndrome coronavirus and currently, the Coronavirus pandemic has caused greater damage to global health [54]. Considerable progress has been made to meet the demand of detection and technological aspects for better progression but despite the advancements, current approaches (counting on culturing techniques and biochemical analysis) haven’t discovered any possible solution to the disease diagnostics and the current methods are time-consuming, and labor demanding [55]. Additionally, the emergence of pathogens that are resistant to current drugs, possess very consequential threats to the health of living beings, and information on antimicrobial susceptibility is critical to developing better therapeutics but current technology makes it longer to get access [56]. To address the pathogenic thread, we need a simple, accurate, cheap, sensitive, rapid, portable, detecting range of pathogens and reliable diagnostic methods to capitalize on the pathogens [56].

The CRISPR-Cas protein is a programmable genetic protein that has the ability to severe the DNA or RNA of the desired organism and configures it to our likes as they identify invading pathogens by their nucleic acid sequence and eventually neutralizes them through cleavage, i.e., their cis and trans-cleavage properties along with their crRNA (CRISPR RNA) that recognizes the target sequence [57]. Using these features DETECTR and SHERLOCK the two CRISPR-based diagnostics methods were developed and the Cas protein of Cas9, Cas12, and Cas13 were all applied for diagnostics, gene editing, and biomarkers purposes [58]. In recent years many CRISPR-based proteins like NASBACC (nucleic acid sequence-based amplification Cas9-based Zika variant), SHERLOCKv2, and HOLMES were presented for their application in the diagnostics of the pathogen [59]. These CRISPR diagnostics methods are well suited for the detection of pathogens and with small twerking this technology can be sued for the estimation of pathogenic levels the future of CRISPR depends on the design of its sensitivity and compact sensors [59] and we have mentioned the detection of CRISPR-based detection methods for selected few bacterial, viral and for COVID-19.

### 4.1. Bacterial

#### 4.1.1. Food Poisoning (*Escherichia coli*, *Salmonella*, *Staphylococcus aureus*)

In today’s world, food security is considered a global concern. The notion of human security development is related to a feeling of a human duty to sustain one’s life according to a worldwide trend pushing us to offer safe nourishment to humans. Consuming uncooked or undercooked foods might expose one to germs that cause food poisoning. Whether they like it or not, residents in certain locations live in an unsanitary environment where they are unwittingly exposed to enteric pathogens or immunologically cross-reacting microorganisms regularly [60,61]. CRISPR-based biosensing facilities provide a new opportunity for improvement in food security by increasing the sensitivity of bacterial nucleic acid detection in food samples at a faster and more accurate pace. Lba Cas12a (Cpf1), a CRISPR system extracted from the *Lachnospiraceae bacterium*, was combined with a hybridization chain reaction (HCR) system and used to develop anelectrochemical biosensor for the recognition of gene markers specific to *Salmonella typhimurium*, a dangerously common bacterium in many food poisoning and other food borne diseases [62,63].

Cas12a-Ddp is another unique nontransferable, ultrasensitive dual detection platform. The round top enables more Cas12a detection solutions to be temporarily stored than the flat cap, allowing for one-pot tests and decreasing aerosol contamination. The microplate reader and ultraviolet vision read the information in dual mode to achieve sensitive dual-target detection of pathogenic *Salmonella* genes and drug resistance genes simultaneously, enabling onsite analysis. Multiple polymerase chain reactions and recombinase polymerase amplification were used to combine Cas12a-Ddp [64]. Without any cross-reaction, a high-sensitivity dual LOD of 1 CFU/mL was reached after 40 min. In liquid matrix meals, the presence of lipids and other proteins didnot greatlyaffect the working Cas12a-Ddp. Thus, with a dual LODof 1 CFU/mL and a spiking recovery of 68.58–158.49%, it was also employed to identify drug-resistant salmonella in milk and skim milk powder [64].

A fluorescence sensing approach for the detection of *E. coli* O157:H7, which is simple to use and efficiently based on the ability of the CRISPR-Cas9 system to recognize and cleave target virulence gene sequences, followed by strand displacement amplification and rolling circle amplification following amplification probes can form hybrids with large products whose fluorescence is quenched using a metal-organic framework platform, resulting in fluorescence recovery at typical excitation/emission wavelengths of 480/518 nm [65,66]. Another technique called CCB-Detection is a bacterial sensing technique that researchers created (CRISPR-Cas13a-based Bacterial Detection). CCB-Detection indicates good selectivity for *S. aureus*, with minimal interference from other bacteria. Furthermore, CCB-Detection was effectively used to detect *S. aureus* in real food samples that contained both known and unknown amounts of bacteria (spiked and non-spiked). Its potency was nearly equivalent to that of the conventional culture-based counting methods, albeit with a shorter cycle time and increased sensitivity [66,67].

#### 4.1.2. Tuberculosis

Tuberculosis is a serious contagious ailment that is caused by bacteria called *Mycobacterium tuberculosis* that substantially affect the lung, but it is curable and preventable. According to WHO, an estimated 10 million people fell ill with tuberculosis (TB) and 1.5 million people died from TB in 2020. Worldwide, the alternate prominent contagious killer after COVID-19 is TB, and TB is the 13th leading cause of death. This statistic shows that *Mycobacterium tuberculosis* has a great impact on global health. Due to the necessity of detecting and diagnosing MTB (*mycobacterium tuberculosis*), the development of genetic tools has occurred for manipulating the MTB so that the characterization of appropriate drug targets can take place [68]. There is a huge diversity of biosensors such as electrochemical biosensors, sandwich antibody-based biosensors, amperometric DNA biosensors, dual-aptamer-based voltammetric biosensors, and CRISPR-based biosensors for the detection of *Mycobacterium tuberculosis*.

*Mycobacterium tuberculosis* is a Gram-positive genus of bacteria associated with Actinobacteria, having a type III-A CRISPR family [69]. This type III-A CRISPR system is classified as the “Mtube” subtype based on the genes present in the Mtube stain, namely, H37Rv [70]. For identifying MTB, the CRISPR-MTB test is evaluated as it is extremely sensitive, and with a near single-copy sensitivity, it needs less sample input and has a shorter turnaround time than Xpert. The diagnostic achievements of the CRISPR-MTB test were correlated in the same direction with culture and Xpert (GeneXpert MTB/RIF assay). The CRISPR-MTB test revealed an enhanced result and boosted overall diagnostic performance, above culture and Xpert, and provides a considerable possibility as a new diagnostic technique for both pulmonary and extra-pulmonary tuberculosis [71].

#### 4.1.3. Chlamydia

Chlamydia is one of the most common sexually transmitted diseases, which is caused by bacteria called *Chlamydia trachomatis*. Both men and women canbe infected by chlamydia, as women can get chlamydia in the cervix, rectum, or throat and men can get chlamydia in the urethra (inside the penis), rectum, or throat. For the very first time, the presence of the CRISPR system was discussed in terms of the order Chlamydiales, and the immune system of bacteria composed of a small CRISPR locus located on the chromosomes involving eight repeats and Cas-Cse genes of the subtype I-E is associated with it. *Chlamydia* sp. Diamant also has a CRISPR locus [72].

In chlamydia, the formation of a solitary plasmid process for CRISPRi is addressed to an unnecessary gene, *incA*, which reveals a gratuitous inclusion of membrane protein. The unflappable alteration of *Chlamydia trachomatis* serovar L2, including plasmids that encode only dCas9, is unsuccessful in averting the expression of *incA* under the domination of an inducible promoter, or simply the guide RNA (gRNA) that targeted the 5′ UTR of *incA*, stated constitutively, as *Chlamydiae* did not possess a deleterious effect of expression of dCas9 alone and has the least impact on chlamydial growth or development. However, the introduction of the expression of dCas9 generated the fluctuating suppression of *incA* expression when the plasmid fused the dCas9and gRNA targeted *incA* [73].

Based on the dCas12 system, the performance of a second CRISPRi system increases the number of potential chromosomal targets. With the help of these tools, the study of essential gene function in chlamydia became possible [74].

#### 4.1.4. *Helicobacter pylori*

*Heliobacter pylori* is a Gram-negative, helical, microaerophilic bacteria that is liable for ailments such as gastric cancer, peptic ulcer, and MALT (mucosa-associated lymphoid tissue) lymphoma. Many diseases are associated with the genes *cagA* (cytotoxin associated gene A) and *vacA* (vacuolating cytotoxin A) of *Helicobacter pylori*. Each gene has its encoding area and the complication associated with it, such as the *cagA* gene, which encodes the oncoprotein of bacteria leading to deregulation of cell growth, irregular cell signaling, and inhibition of cell-cell contact. Elongated epithelial cells and toxin that forms pores are encoded by vacA, which prompts disruption of endosomes, inhibiting leukocytes by vacuolization, and inducing cell apoptosis (epithelial) [75]. It has been reported that *cagA* and *vacA* are directly related to the formation of gastric cancer and the formation of ulcers [76,77]. *H. pylori* has shown recombination properties and a high level of mutations, allowing the generation of new alleles. *H. pylori* has established itself as an efficient bacterium owing to its quick adaptation in areas such as the stomach and duodenum, and various mutated strains were found to be living in the gastric area [78]. *H. pylori* has shown that it has vast genetic diversity, starting from genetic mosaicism to polymorphisms of nucleotides; this was found through analysis of its genome [79].

Invasive and non-invasive strategic methods are the two most widely diagnosed strategies that have been used for the prognosis of *H. pylori* infection. Endoscopic techniques are performed in the invasive test methods, whereas non-invasive test methods comprise the urea breath test, the rapid urease test, the antigen test, and microbial culture. Serological tests(detects IgG) are largely applied for testing purposes [80]. The commonly used non-invasive methods are not reliable and have limitations. *H. pylori*, which is present in the oral environment, possess a problem with the urea breath test, as it can interfere, and if the test is taken for children, then inadequate carbon dioxide production tampers the test [81,82]. Even after the treatment of *H. pylori*, their antibodies can exist in the circulatory system for a long timeafter eradication, which may lead to false-positive test results, provoking unwanted treatments [83,84]. It is also reported that a few laboratories make use of PCR to detect *H. pylori* in stool samples; however, it has thus far been challenging, and also a few substances from samples may hinder the PCR assay [85]. These non-invasive methods are non-confirmatory, are less accurate for diagnosis of bacteria, and cannot be applied for the screening of infected patients on a large-scale; consequently, the association of endoscopic methods accompanied by histological studies is more precise for *H. pylori* detection. Nevertheless, these methods are expensive [86].

To improve the quality of the detection methods, a novel, rapid, easy to access, accurate, and lighter device was required, and biosensor technology emerged as the affordable method to detect *H. pylori*, as biosensors can analyze pathogenic samples by uniting with the bioreceptor (recognizing the biological part) with a transducer that converts its biological presence into a finite signal that is either electrical or optical [87]. Importantly, preparation of samples is not needed to measure analytes by utilizing a biosensing device, and delivering accurate results and real-time tracking of diseases have promoted the usage of biosensor technology in every aspect of science and technology [88]. In recent decades, various biosensor-based detection techniques have been developed to improve the existing technology; some biosensors include a fluorescent DNA biosensor for rapid detection of *H. pylori* DNA [89], and a voltammetric-based biosensor that utilizes a bismuth-immobilized carbon nanotube electrode (BCNE), which was also developed to detect *H. pylori* [90]. Various DNA-based biosensors have been developed, including a colorimetric paper device developed by Monsur Labs, which is DNAzyme-based and is simple and capable of providing specific and sensitive detection of *H. pylori* [91]. Phendione has been used as an electrochemical ligand in a DNA biosensor to detect *H. pylori* [92]. Using Oracet blue (OB) and a gold electrode (AuE), an electrochemical DNA biosensor was developed to detect *H. pylori* [93]. A urea biosensor was also developed that utilizes the urease enzyme that was obtained from *Helicobacter pylori* [94].

Recently, CRISPR technology has been extensively applied to detect infectious diseases [95], and a CRISPR-based detection method was evaluated by Enming et al. to rapidly detect *H. pylori* in stool samples; they used the CRISPR-Cas12a protein for the detection process in conjunction with a gold nanoparticle (AuNP)-based lateral flow biosensor (LFB), which was used to display the detection result. The LFB has helped to reduce the necessity of knowledge required to interpret the results. In this detection method, RPA can isothermally amplify the chosen DNA that was merged with the CRISPR-Cas12a protein for detecting, and agarose gel electrophoresis was used to optimize the primer for RPA along with a fluorescence signal to detect the best coupled gRNA with the Cas protein. The detection limit was then established through the fluorescence-CRISPR assay and LFB-CRISPR assay [96]. The LOD was measured at 5 copies/μL, and this recent methodology was tested for accuracy and sensitivity along with other common gut pathogens that include *Vibrio parahaemolyticus*, *Enterococcus faecalis*, *Shigella sonnet*, *Escherichia coli*, *Staphylococcus aureus*, *Staphylococcus epidermidis*, *Shigella flexneri*, and *Salmonella enterica*; these results indicate that the CRISPR-Cas diagnostic method achieved a good performance level [97]. Here the usage of Cas12a protein is preferred over Cas13a and Cas9 due to simple preparation methods and usage of one crRNA; therefore, Cas12a was established as a better Cas protein than others, and also the usage of LFB for displaying the detection result makes it feasible for its use in POCT for extensive screening studies and epidemiological studies [97]. Although the CRISPR system is widely used in a wide range of research fields, its usage for the detection of *Helicobacter pylori* is limited, and even though various biosensors are used for detection, a wholly CRISPR-based biosensor is needed for *H. pylori* detection.

### 4.2. Viral

#### 4.2.1. Hepatitis

In recent years, research development on hepatitis virus has been actively carried out, and its types—hepatitisA, B, C, D, E, and G—have shown developments in diagnosis, epidemiology, drugs, clinical aspects, and molecular features, and vaccines are available for hepatitis A and B that have been reported to cause frequent infection in children [98,99]. According to the World Health Organization, every type of hepatitis causes liver disease, and to be specific, hepatitis B and C are more infectious and threatening, as they are the common cause of hepatocellular carcinoma, liver cirrhosis, liver fibrosis, liver cancer, and hepatitis-affiliated deaths, and an estimation of 354 million people worldwide live with hepatitis B or C; most importantly, hepatitis C does not have an availability yet [100]. Hepatitis virus transmits through human blood; thus, the importance of detecting the virus in an early stage is a vital part to eradicate and cure it [101]. The conventional method to detect any hepatitis-type strain includes loop-mediated isothermal amplification (LAMP), polymerase chain reaction (PCR), and immunoassay based on serological methods, etc., which are time-consuming, labor-demanding, expensive, and provide false-positive signals; all these factors have contributed to prevent cost-effective, high sensitive clinical applications [102]. To provide, accurate, labor-free, efficient detection techniques, biosensors have been used for the detection of the hepatitis virus, and a few of them include electrochemical DNA biosensors to detect hepatitis A [103], hepatitis B-based plasmonic biosensors [104], MEMS cantilever biosensors for the detection of hepatitis C and A virus [105], and an electrochemical biosensor based on the electrical pulse to detect hepatitis E [106]. An electrochemical immunosensor array was designed and developed for simultaneous detection of five types of hepatitis, namely, A, B, C, D, and E, paving the way for a multi-analyte detection system for point-of-care testing [107]. The advent of CRISPR-Cas genome editing technology has proven its potential for the evolution of next-generation-based diagnostic methods due to its capability of providing high specificity, reliability, and sensitivity [26], but currently, CRISPR-based biosensors for the detection of hepatitis virus are only available for hepatitis B. CRISPR provides a more promising approach for detection, owing to its highly efficient sensitivity and specificity [107].

Chen et al. integrated a lateral flow biosensor that is gold nanoparticle-based merging with the CRISPR-Cas protein. PCR is preamplified in order to increase the sensitivity of CRISPR-Cas and, using a nucleic acid amplification technique known as multiple cross displacement amplification (MCDA), an alternative for PCR to be used in point-of-care, led to the development of an MCDA pre-amplification step with the CRISPR-Cas12b-LFB readout. “CRISPR-HBV”was developed, a novel rapid detection technique that is ultrasensitive and highly specific to detection of HBV (along with genotypes B and C) [108]. The CRISPR-HBV test possesses a high degree of specificity to detect and also to distinguish HBV genotypes (B and C), and to meet the demand for primer design, an MCDA-D1 primer with the PAM site (TTC) of CRISPR-Cas12b was modified. The gold nanomaterials used in LFB are becoming the go-to materials, owing to their biocompatibility, high adsorption, high surface-to-volume ratio, simple synthesis, and manipulation. This detection process of this novel CRISPR-HBV assay can be completed in an hour and can detect 10 copies of DNA/test without reacting with other agents, having great potential to be used in POCT [108].

The colorimetric method of detection is of immense significance for point-of-care testing, and Shaohua et al. developed an SDACC method (strand displacement amplification assisted CRISPR-Cas12a) based on colorimetric analysis for the detection of HBV DNA. The strand displacement amplification (SDA) method was used to generate abundant single-strand DNA (ssDNA) by the help of the target, which is hepatitis B virus, and this strategy can identify mismatched DNA sequence and can detect HBV DNA; this process can bypass the complicated amplification and detection tasks, and this process is a low-cost, sensitive colorimetric detection method to be used in viral diagnosis [109].

CRISPR-Cas13a was used in the detection of HBV DNA, where CRISPR-Cas13a was combined with the conventional PCR amplification technique to detect low-level hepatitis B DNA; the PCR-CRISP assay showed extremely high sensitivity to detect HBV DNA, and since PCR amplification is considered more stable, it was used before CRISPR-Cas13a. By combining the conventional PCR with CRISPR-Cas13a, the sensitivity was increased threefold, as this method has experimented with ddPCR and qPCR, and their sensitivity turned out to be lower than PCR; this method can also be used for screening for HBV virus before blood transfusion and organ transplantation [110].

Surface-enhanced Raman scattering (SERS) is a powerful technique developed from Raman spectroscopy to identify the chemical structure and measure biomolecules in low concentrations, and they are excellent in sensitive detection with optimized nano-materials, and using this, Jin-ha et al. developed a viral DNA biosensor that is amplification-free along with the SERS analytical device aided by CRISPR-Cas12a. This integration of nanomaterials led to the development of an ultrasensitive system where there is an omission of nucleic-acid amplification, thereby maximizing the intensity of SERS signals; this system proved to have a low LOD, short reaction time, multiple detections, and a wide linear dynamic range (LDR). Hepatitis B virus (HBV) was used as the target DNA, and this proved fruitful, as an ultra-high level of sensitivity and selectivity was observed; this system also has extraordinary adaptability and should be employed in POCT [111].

A LAMP-Cas12a biosensor was developed to solve the problem of nucleic acid extraction. LAMP was used to replace the poor amplification by recombinant polymerase isothermal amplification (RPA), the longer amplification time of PCR, and providing the desired result in short span of time. Inherent HBV viral DNA was extracted from the sample, and after pre-processing were amplified by LAMP and mixed with Cas12a-crRNA to detect the viral sequence. The presence of HBV DNA activates the properties of CRISPR-Cas12a, thereby cleaving the fluorescent reporter and releasing the fluorescent signal. The output to read this signal could be read either by fluorescent readout or by using lateral flow strip technology, enabling the test to be viewed under direct eye; 100% specificity and sensitivity for both fluorescent readout and lateral flow test strip were found [111]. It is also worth mentioning that with 1 μL of HBV it is possible to have HBV detection in the fluorescent readout within 13 min, and lateral flow test strip takes 20 min for detection. With minimal equipment, low-cost requirements, and rapid and accurate detection, this biosensing technology adds significant value to the POCT system to detect HBV virus [111].

#### 4.2.2. Human Immunodeficiency Virus (HIV)

HIVis a virus that targets and harms the immune system by damaging the white blood cells. At the end of 2020, it was estimated that 37.7 million people were living with HIV. According to WHO, 1.5 million (1.0–2.0 million) people acquired HIV, and 680,000 (480,000–1.0 million) people died from HIV-related causes. CRISPR helps in genome editing at the DNA level, and CRISPR-based tools provide new prospects to inhibit HIV, a retrovirus that uses both RNA and DNA that are forms of genetic information [112]. CRISPR technology is presumed to play a vital role in this context as well as use as a sensor against viral infections and is currently an emerging field [112].

The provirus can be targeted by one or more small guide RNAs (sgRNAs) conjugated with Cas9 nuclease to mediate the excision of the integrated viral genome. By using sgRNAs, HIV excision mediated by CRISPR-Cas9 prevailed and targeted preserved sites inside the U3 region of the HIV-1 LTR in a number of latently uninfected microglial, promonocytic, and T cell lines. Viral reactivation and replication in these infected cells can be prevented by using two sgRNAs synchronously and inducing highly efficient editing. Dual sgRNAs helped the cells to be pretreated and successfully targeted non-integrating virus by offering much tougher evidence for blocking the integration of viral DNA, and as de novoHIV infection was resisted by CRISPR-Cas9 [113]. Cas9-induced DNA splitting is generated in the PAM-proximal sequence area that is crucial for gRNA strapping and target DNA splitting, whereas Cas12a-provoked DNA cleavage is generated in the distant area of the PAM sequence that is lower, which ispivotal for target binding and splitting. This is an important characteristic of Cas12a for gene deactivation, as the amended DNA sequence—cleaved and mended with the regular participation of interpolation or elision(also called indel)—can likely be retargeted and composed as an ampler indel type of mutation benignant for maximum HIV provirus deactivation, and it may avert the generation of fleeing virus variants along with a minimum mutation, while HIV genomes with a greater dramatic mutation are more presumably to be replication-incapable. Hence, Cas12a is known as a supercilious anti-HIV tool despite Cas9 [113,114]. Moreover, HIV replicates in cells to generate DNA from the viral RNA genome by using reverse transcriptase enzyme and viral RNA expression from the activated latent HIV-1 DNA suppressed by Cas13a, which is a powerfulantiviral technology that inhibits a wide assortment of RNA viruses [114].

#### 4.2.3. Dengue

Dengue is a mosquito-borne viral infection. The main source of dengue transmission is the mosquito vector that is Aedes aegypti. Dengue virus (DENV) is called a single-stranded positive-sense RNA virus, and it is associated with the genus Flavivirus within the family Flaviviridae [115]. There are four serotypes of the Dengue virus, namely, (DENV-1, DENV-2, DENV-3, and DENV-4), and they are spread throughout America and in some countries.

The CRISPR-Cas9 system depends on RNA-DNA base-pairing, which is used to produce site-specific mutations in *Ae. aegypti* and targeting specificity that results in effective and flexible genome-editing reagents. CRISPR–Cas9 is an effective, unbiased, and reproducible way to recognize important host factors in flavivirus infections, as flavivirus includes DENV and can produce distinctive kinds of mutations through disparate repair mechanisms, as well as outline the stable germline mutations in several genomic loci. A pooled group of cells is infected by flavivirus involving DENV, in which Cas9 along with whole genome gRNAs have been proposed and cultured together for the selection of rare mutant cells to inhibit viral entry, translation, replication, or cell death induced by virus under highly strict conditions [116,117]. A novel and efficient method of CRISPR known as CRISPR13a is considered to inhibit Dengue viral replication [117]. Table 1 shows the used amplification technique along with the Limit of Detection (LOD) for pathogens detect by the CRISPR-Cas system.

#### 4.2.4. Human Papillomavirus (HPV)

Human papillomavirus (HPV) infectionis the most prevalent trigger of cervical cancer in females. Cervical cancer claimed the lives of roughly 311,000 women in 2018, with about 85% of these casualties occurring in countries with low to average incomes. Cervical carcinoma hasthe second highest prevalence of cancer in women worldwide, with human papillomaviruses responsible for more than 99 percent of cases (HPVs). HPVs, particularly the high-risk types (HR-HPVs), must be detected early to prevent disease progression [118]. For decades, cytology-based detection has been a mainstay for the prevention of cervical cancer. However, it necessitates the upkeep of complicated infrastructure and highly trained workers as well as relatively short screening intervals to ensure accuracy [118]. Because of its low specificity, conventional HPV-based screening should be avoided in females under the age of 30, and in some cases, under the age of 35. Furthermore, within a cervical cancer screening and treatment program, a positive HPV test result demands adequate assessment, recommendation, and repeated testing using a well-defined procedure. Most countries use cytology as a triage test after a positive HPV screening result, avoiding direct referral to colposcopy; future therapy is contingent upon the cytology result as to whether the patient will be referred to colposcopy or repeat testing [118]. Another significant difficulty is the large number of commercial HPV assays on the market, which makes selecting the optimum test for cervical cancer screening programs difficult. Since there are no meaningful performance assessments in the peer-reviewed literature, the vast number of commercially marketed HPV tests remain inappropriate for testing. Instead of this lack of screening facilities, a new generation of CRISPR Cas-based biosensors is in the development pipeline, most of which are focused on Cas12a-based CRISPR systems. Some of these development efforts are mentioned in this article.

A non-genotyping technique for sensing 13 kinds of HR-HPVs utilizing the RPA-Cas12a system was proposed, enabling the determination of an individual’s HR-HPV infection status. The test comprised nucleic acid amplification using RPA on a primer pool generated from the GMY/GP6+ primer set, followed by detection using CRISPR-Cas12a. This developed test was quick and required little equipment; its use aided in the traditional procedure for the testing of cervical cancer [118].

Using the E-CRISPR platform, several biosensing techniques were achieved for detecting HPV viral DNA. After enhancing the trans-cleavage activity of the Cas12a complex used in the E-CRISPR platform invitro, it was used to recognize viral nucleic acids in particular sequences related to HPV-16 andPB-19, with sensitivity in the order of picomoles [119]. Researchers developed a straightforward, flexible, electric field-enhanced (EFE), electrochemical CRISPR biosensor to identify target DNA sequences in a homogeneous solution phase. To improve sensor performance, a pulsing electric field was employed to aggregate nucleic acids on the surface of the electrode. The EFE electrochemical CRISPR biosensor detects electrochemical DNA even without the requirement for sophisticated electrochemical probe immobilization by directing the diffusivity difference between electrochemical oligonucleotide probes and CRISPR-cleaved probes onto a negatively charged working electrode. This CRISPR biosensor was able to detectHPV-16 DNA directly without the need for amplification up to a sensitivity of 1 pM. Additionally, the developed biosensor could detect HPV-16 genes in clinical specimens when used in conjunction with RPA [120].

Furthermore, a CRISPR-Cas12a system designed for HPV DNA detection, integrated into lateral-flow strips, was able to recognize HPV-18 and HPV-16 gene markers directly and specifically in liquid matrix samples, with a LOD of 0.24 fM, which is similar to PCR-based sensors but far more rapid. Furthermore, HPV16 and HPV18 were detected in plasma samples from 13 of 14 and 3 of 10 individuals with histological cervical cancer diagnoses using this method [121].

**Table 1:** Overview of detection of pathogens along with the Cas proteins.

Pathogen	CRISPR	Nucleic Acid	Amplification	Readout	LOD	Detection Platform	Ref.
Food poisoning (*E. coli*, *Salmonella*, *S. aureus*)	Cas9a	DNA	SDA	Colorimetric	100 copies	-	[122]
	Cas12a	dsDNA	HCR	Electrochemical	20 CFU/mL		[122]
	Cas12a-Ddp	dsDNA	PCR	Microplate	1 CFU/mL	Dual detection platform	[122]
	Cas13a	ssRNA	PCR	Florescence	1 CFU/mL	CCB-Detection	[122]
Tuberculosis	dCas9	DNA	PCR	Bioluminescence	≈3 × 10^−21^ M	Chimeric dCas9-luciferase	[123]
	Cas12a	DNA	RPA	Florescence	1 copy	CRISPR-MTB	[123]
*H. pylori*	Cas12a	DNA/RNA	RPA	Lateral flow strips, visualization	5 copies/μL	Lateral flow biosensor	[124]
Hepatitis Liver cancer	Cas12a	DNA/RNA	HCR	Gel electrophoresis	1.5 fM	-	[125]
	Cas12a	DNA/RNA	LAMP	Colorimetric	10 aM	AuNP colorimetric	[126]
	Cas12a	DNA	-	Colorimetric	10 pM	MAV-chip	[127]
HIV	dCas9	DNA	-	Fluorescence	-	Nanoelectrokinetic chip	[128]
	Cas12a	DNA	-	Resistive pulse	10 nM	Nanopore sensor (SCAN)	[129]
Zika/Dengue Virus Zika	Cas9 (NASBACC)	RNA	NASBA	Colorimetric	1 fM of RNA amplicon	NASBACC	[130]
	Cas13a	DNA	RPA	Fluorescent	9 aM	Droplet microfluidics	[131]
	Cas13a	RNA	RT-RPA	Fluorescent/colorimetric	0.9 aM in saliva	Lateral flow assay	[132]
HPV16/18	Cas9 (CARP)	DNA	PCR	Electrophoresis/qPCR instruments	2 pg of DNA amplicon	CARP	[133]
	Cas12a (DETECTR)	DNA	RPA	Fluorescence	ca. aM of viral DNA	DETECTR	[133]
	Cas12a	DNA	RPA	Colorimetric	0.24 fM	Lateral flow assay	[133]
	AaCas12b	DNA	RPA	Fluorescence	1 × 10^−18^ M	CDetection	[134]
	Cas12a	DNA, Protein	-	Electrochemical	10^−12^ M	E-CRISPR	[134]
	Cas9	DNA	qPCR	qPCR	2 ng	ctPCR3.0	[135]
	Cas12a	DNA	RPA	Fluorescent	10−100 copies	One-pot reaction	[136]

LOD: Limit of detection.

## 5. SARS-CoV-2 (Severe Acute Respiratory Syndrome Coronavirus 2)

The 2019 pandemic-causing coronavirus disease, also known as COVID-19 (SARS-CoV-2), was initially detected in China on 31 December 2019. SARS-CoV-2 spread rapidly over the world, prompting the World Health Organization (WHO) to proclaim on 30 January 2020, that the COVID-19 outbreak was a public health emergency of international significance [137]. The major routes of transmission are respiratory droplets; SARS-CoV-2 can be transmitted to a healthy person if they comes into contact with an infected person or any of their things, such as clothes, doorknobs, etc. Aerosol transmission (airborne transmission) of this virus has also been observed in research studies [137]. Individuals infected with a novel variant ofSARS-CoV-2 have a wide range of symptoms, ranging from asymptomatic to acute respiratory distress syndrome and multi-organ failure. As a result, diagnosing COVID-19 accurately has proven to be difficult [138].

**Figure 3:**
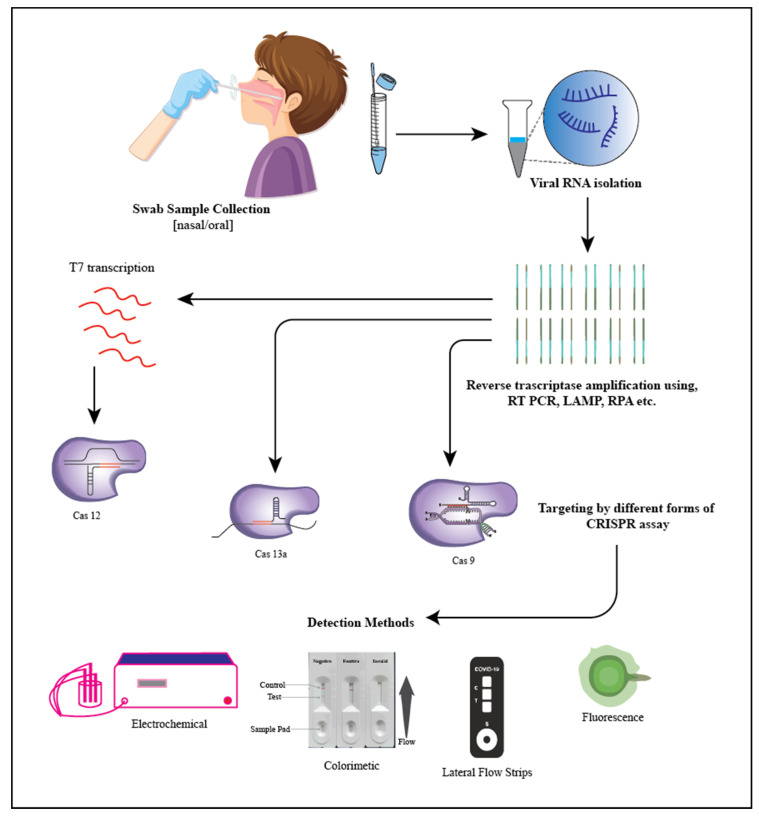
Five-step workflow of CRISPR-based detection system to detect SARS-CoV-2 virus. (1) Sample collection using nasal or oral swabs. (2) RNA molecules are extracted and isolated from the sample, as SARS-CoV-2 is an RNA-based virus. (3) Isolation is followed by an amplification procedure such as RT-PCT, RT-RPA, or RT-LAMP to increase the LOD, (4) followed by detection using the CRISPR-Cas system and (5) detection using fluorescence, colorimetry, electrochemical, or other sensory readout methods.

COVID-19 has so far been diagnosed using associated symptom analysis anda variety of laboratory detection methods, which include nucleic acid amplification tests (NAAT), computed tomography (CT) scans, and serological procedures [139]. Molecular and serological testing are the two types of analytical diagnostic procedures for COVID-19 tests that are currently accessible and Figure 3 depicts the methodology on detecting COVID-19 using a CRISPR-based detection system. The reverse transcription-polymerase chain reaction (RT-PCR)-based approach is used in the first category of molecular tests for the detection of SARS-CoV-2 virus RNA. Other approaches, such as isothermal nucleic acid amplification tests, transcription-mediated amplification, and CRISPR-based technologies, offer potential alternatives to RT-PCR testing. Here we mostly review alternative PCR technologies that involve the use of CRISPR Cas systems [140].

The samples are gathered from the patients in the first stage. A healthcare professional could collect them using Dacron or polyester flocked swabs. For SARS-CoV-2 detection, saliva specimens have recently been added as something of an alternative sample source [141].

Viral RNAs must be isolated from the raw sample once it has been collected. RNA isolation processes usually consist of three steps: sample cell lysis; RNA isolation from other biomolecules such as DNA, proteins, and lipids; and finally RNA elution [142]. One of these three major devices can be used to separate viral RNA molecules.

1. Spin column-based NAE (nucleic acid extraction) provided the most suitable option for RNA isolation. It plays an important role in ion exchange methods because it offers a stable stationary phase for fast and reliable buffer exchange [142].
2. Purification with magnetic beads. Magnetic beads are used to collect viral RNA in this procedure. During the wash and collection, the beads are held in place by an external magnetic field. The magnetic format enables quick sample collection and concentration. Manually capturing and releasing particles, on the other hand, might be time-consuming [143].
3. Extracting organically. In this method, a phenol-containing solution is used to homogenize the specimens, which are then centrifuged. The viral RNA is found in the top aqueous phase during centrifugation. The viral RNA is isolated from the upper aqueous phase and recovered during centrifugation, precipitating it using alcohol (elution) and later placing it in a rehydrating medium. This approach is regarded as the finest for RNA extraction; nevertheless, it is tedious, time-consuming, and difficult to automate [143].

RNA isolation is usually followed by an amplification procedure such as RT PCT, RT RPA, or RT-LAMP to increase the LOD. Following that, the Cas-CRISPR RNA (crRNA) complex will detect a particular area of SARS-CoV-2. The amplification primers and crRNAs should target the same SARS-CoV-2 locus. Fluorescence- or colorimetric-based devices were the most frequent techniques for signal reading. The CRISPR assay may be included in lateral flow readout strips as well [144]. So far, the vast majority of tests developed have used the Cas12 or Cas13 family of CRISPR proteins as CRISPR effectors [144]. Table 2 shows the CRISPR protein that is capable to detect COVID-19.

Firstly, the intended amplicons are added to the Cas12a/crRNA complex in the Cas12a assay. Then after binding to the intended RNA-guided target, the Cas12a complex undertakes collateral cleavage of the region surrounding the non-target reporters. Single-stranded DNA that has been fluorophore quencher (FQ) or fluorophore biotin (FB) labeled is commonly used as a reporter. The signal readout can be done as a fluorescence-based reaction or a single-plex colorimetric lateral flow reaction after the non-targeted ssDNA reporter has been trans-cleaved [144]. With a few minor differences, the procedure for the Cas13 assay is nearly comparable to the Cas12a-based technique. Because RNA targets would only activate Cas13a proteins, following amplification, an extra T7 transcription is required to convert the DNA amplicons to RNAs. Fortunately, the amplification and Cas13a test may be combined into a single-pot reaction. Furthermore, because activated Cas13a cleaves ssRNA rather than ssDNA, ssRNA reporters should be employed rather than ssDNA reporters in Cas12a systems [144].

Moon et al. Recently a viral detection approach with a colorimetric read-out mode based on the clustered regularly interspaced short palindromic repeats (CRISPR)-Cas9 endonuclease dead (dCas9) system was developed [144]. The CRISPR-dCas9 system was used to directly detect RNA in the viral lysate using a biotin-protospacer adjacent motif (PAM)-presenting oligonucleotide (PAMmer). The color shift was caused by the oxidation of 3,3߰,5,5߰-tetramethylbenzidine by streptavidin-horseradish peroxidase linked to biotin-PAMmer. They were able to visually identify SARS-CoV-2, pH1N1, and pH1N1/H275Y viruses using the established approach.

## 6. Future Prospects

Timely detection using rapid technology and assessing the correctinfection are keys to effectively implementing control and eradication of any prevailing infections or diseases. To improve the detection and control of diseases, effective diagnostics technology is required, and current technology has major issues surrounding them, such as cost, sensitivity issues, higher detection time, etc. The emergence of CRISPR-Cas sensor technology has the potential to overcome a few obstacles to show reliable results [156]. CRISPR-Cas is a highly specific biological tool that cleaves the target DNA/RNA, thus having high accuracy, sensitivity, and specificity. This provides better performance than other methods, as this system is superior to the existing methods of detection of pathogenic organisms’ DNA and RNA, thereby proving their stronghold on the point-of-care system (POC) [156].

Different CRISPR-Cas tools have shown different properties towards diagnostics. For example, C Detection (Cas12b) was shown to differentiate bases individually, thus providing a platform that can identify single bases. When comparing between Cas12 and Cas14, it was shown that Cas14 was able to distinguish a single nucleotide polymorphism (SNP) responsible for changes in eye color, but Cas12 failed to recognize this. The CRISPR-Cas technology has progressed from a simpler cleaving mechanism to integrating the CRISPR protein for gene editing, developing a simple biosensor, and diagnostics methods, and this suggests CRISPR as the future of biotechnology and its allied areas due to its properties, as it can provide an answer for fundamental and applied research areas [156]. It is worth noting that CRISPR-Cas sensing technology has solved the low sensitivity issue that is faced by current detection methods [157]. For instance, a CRISPR-based diagnostic was able to detect attomolar quantities of foreign particles, showing their higher sensitivity when compared with PCR-based methods with a femtomolar range of detection. CRISPR-based techniques have multiple applications in areas of medicine and beyond; e.g., they are used to detect the genotypes of pathogenic organisms to provide specific medication to them [158]. Detecting a specific pathogen allows for the rapid evaluation of treatments. Another important characteristic of CRISPR is that we can detect any nucleic acid sequence just by changing the gRNA sequence; thereby, we can detect pathogens from any range of organisms [158].

However, despite all the advances talked about, most of the CRISPR-Cas sensors that have been published are designed to detect nucleic acids and associated targets. As a result, there is still a need for new methods for the development of activatable CRISPR sensors that can be applied to a far broader spectrum of targets other than nucleic acids. Several challenges include oversensitivity, off-target recognition, and false positives arising from the intrinsic capacity of the CRISPR system to tolerate mismatched nucleotides, which hamper the rapid emergence of CRISPR-based biosensors [158]. Moreover, aside from SHERLOCK, which was recently approved by the FDA in 2020 for use as an emergency alternative for the diagnosis of SARS-CoV-2 in the USA, most other POCT solutions discussed in the scope of this article have not yet been widely applied in the healthcare system when seen in the light of the use of RT-PCR and similar conventional approaches. Further development and improvement of these CRISPR detection methods remain necessary for broader and greater practical applications in POCT settings [158,159].

The majority of CRISPR biosensors is focused on the detection of nucleic acid, while the detection of non-nucleic acid faces a major challenge due to the collateral cleavage property of the Cas protein that requires a method to transduce the target recognition, which is activated by the binding activity of crRNA to its DNA, and even though a solution involving binding activities of allosteric transcription factors (TFs) of bacteria to detect dsDNA and small molecules has been developed, it is still limited [159]. Additionally, sample pre-treatment is an important aspect when considering nucleic acid testing, which includes food testing and manually performing POC applications; currently, every biosensor based on CRISPR requires pre-treatment of the raw sample to the screen-out virus; to solve this issue, HUDSON was developed that allows for the amplification of the target instantly after heating it, but the sensitivity of detection is lower than methods used for RNA/DNA detection; therefore, in the future, robust Casprotein is required to solve this problem [159]. Another persistent problem with CRISPR is that in the case of in vivo detection using CRISPR, the complex nature of chromosomes and the cellular environment of the cell make CRISPR produce low searching efficiency. To solve this a directing tool needs to be developed that can help CRISPR move in the nucleus; however, this method is still in its infancy stage. The advance toward CRISPR-Cas-based biosensing technology that is rapid, portable, fast, reliable, and compatible is steady. We expect CRISPR technology to further revolutionize sensing strategies, thereby leading to the development of POCT devices globally [159].

## 7. Conclusions

The need for rapid, reliable, and easy access detection methods has paved the way for the CRISPR-based biosensor to be inducted in POCT. CRISPR biosensors have great sensitivity, shorter detection cycles, and are cheap, making them the choice of instruments suited for emergencies. To solve the accuracy problem faced by the electrochemical sensor, it was integrated with the CRISPR-Cas12a system to create E-CRISPR, which is robust and portable. Although E-CRISPR is reliable, it also comes with a few challenges such as low electrochemical response and cleavage efficacy, which are being addressed. The CRIPSR-based colorimetry biosensor was designed to generate a visible signal without the requirement of heavy equipment, thus reducing the cost of its utilization and increasing the methodto detect diseases. The fluorescent sensor is a crucial device that is capable of detecting cancerous cells; so as to create a quick method of reading the results, this sensor was paired with CRISPR-Cas 12a and CRISPR-Cas13 to create powerful and economical detection platforms known as SHERLOCK and DETECTR, whichutilize the power of PAM and crRNA from their respective Cas proteins. DETECTR has enabled rapid and accurate identification of samples, and SHERLOCK was found to detect cancer mutations.

The development and infectivity rate of bacterial and viral organisms have been on the rise in the past decade, and along with this, the continuous adaptability of these organisms towards healthcare procedures enables humankind to invent innovative techniques. The uniqueness of Lba Cas12a (Cpf1), a Cas protein, is that it can detect food-borne pathogens such as *Salmonella typhimurium*, *Staphylococcus aureus*, and *Escherichia coli*. With this foundation, this CRISPR was combined with an electrochemical sensor to create an ultrasensitive dual detection technology that can detect this organism. The conventional platforms used to detect tuberculosis are extrapulmonary TB, pediatric TB, and other paucibacillary TB. The weakness associated with them are lower sensitivity and higher turnaround time, which were solved by the CRISPR-MTB sensor with extreme sensitivity, less sample input, and shorter turnaround time. Additionally, the future of CRISPR-based TB detection is being developed towards automation combined with integrated nucleic acid extraction and examination. These are only possible due to the flexibility of CRISPR-Cas genes. This flexibility has allowed for the design of the dCas12 system to detect *Chlamydia trachomatis*, AuNPs-LFB-based CRISPR-Cas12a to detect *Heliobacter pylori*, CRISPR-Cas12b-LFB readout to detect hepatitis infection, and the Cas9 sensor to detect HIV and dengue. To detect the notorious HPV, aCRISPR-Cas12a system was designed that provides rapid results. The COVID-19 pandemic accelerated the move towards rapid development of CRISPR-based diagnostics units, with over 15 different sensors developed to detect SARS-CoV-2. The use of CRISPR-based techniques to identify and qualitatively assess infectious pathogens may become a new reality in the area of molecular medicine. Creating new CRISPR-based tools and platforms for molecular diagnosis ensures that healthcare management will be reshaped, and medical specialty management will improve on a global scale.

Although CRISPR provides a new toolbox to broaden bioanalytical and biomedical applications, there are challenges; e.g., CRISPR-based detection with isothermal pre-amplification remains unanswered. The current research must focus on portable POC devices that are affordable. Therefore, in the future, these detection devices will be made in a short amount of time and used for the public.

## Figures and Tables

**Table 2:** Overview of CRISPR protein that can be used to detect SARS-CoV-2 virus.

CRISPR Effector Protein	Targeted Gene	Preamplification Method	Signal Readout	Assay Reaction Time	LOD	Reference
Cas9a	N1, N2, and N3 genes	RT-PCR	Colorimetric	Not reported	140 pM	[145]
Cas12a	ORF1ab	RT-RPA	Colorimetric and fluorescence	1 h	10 copies/μL	[146]
Cas12a	N gene	RT-RPA	Fluorescence	40 min	10 copies/μL	[147]
Cas12a	N gene and E gene	RT-LAMP	Colorimetric	30–40 min	10 copies/μL	[148]
Cas12a and Cas12b	N gene and E gene	RT-LAMP	Fluorescence	40 min	10 copies/μL	[149]
Cas12b	N gene	RT-RAA	Colorimetric and fluorescence	1 h	10 copies/μL	[150]
Cas13a	N gene	RT-PCR	Colorimetric	Not reported	10 copies/μL	[151]
Cas13a	ORF1ab and N	RT-RPA	Fluorescence	40 min	1 copy/μL	[152]
Cas13a	Orf1ab, S, and N	RT-RPA	Colorimetric and fluorescence	35–70 min	10 copies/μL	[153]
Cas13a	Not reported	RT-RPA	Colorimetric	50 min	10 copies/μL	[154]
Cas13a	ORF1a	RT-RPA	Colorimetric and fluorescence	1 h	10 copies/μL	[155]

## Data Availability

Not applicable.

## References

[1] Price C.P. (2001). Regular review: Point of care testing. BMJ.

[2] Louie R.F., Tang Z., Shelby M.D.G., Kost G.J. (2000). Point-of-Care Testing: Millennium Technology for Critical Care. Lab. Med..

[3] Bonini A., Poma N., Vivaldi F., Kirchhain A., Salvo P., Bottai D., Tavanti A., Di Francesco F. (2021). Advances in biosensing: The CRISPR/Cas system as a new powerful tool for the detection of nucleic acids. J. Pharm. Biomed. Anal..

[4] Chen Y., Shi Y., Chen Y., Yang Z., Wu H., Zhou Z., Li J., Ping J., He L., Shen H. (2020). Contamination-free visual detection of SARS-CoV-2 with CRISPR/Cas12a: A promising method in the point-of-care detection. Biosens. Bioelectron..

[5] Li Z., Zhao W., Ma S., Li Z., Yao Y., Fei T. (2021). A chemical-enhanced system for CRISPR-Based nucleic acid detection. Biosens. Bioelectron..

[6] Xu W., Jin T., Dai Y., Liu C.C. (2020). Surpassing the detection limit and accuracy of the electrochemical DNA sensor through the application of CRISPR Cas systems. Biosens. Bioelectron..

[7] Qing M., Chen S.L., Sun Z., Fan Y., Luo H.Q., Li N.B. (2021). Universal and Programmable Rolling Circle Amplification-CRISPR/Cas12a-Mediated Immobilization-Free Electrochemical Biosensor. Anal. Chem..

[8] Yao R., Liu D., Jia X., Zheng Y., Liu W., Xiao Y. (2018). CRISPR-Cas9/Cas12a biotechnology and application in bacteria. Synth. Syst. Biotechnol..

[9] Paul B., Montoya G. (2020). CRISPR-Cas12a: Functional overview and applications. Biomed. J..

[10] Hinge V.R., Chavhan R.L., Kale S.P., Suprasanna P., Kadam U.S. (2021). Engineering Resistance Against Viruses in Field Crops Using CRISPRCas9. Curr. Genom..

[11] Kostyusheva A., Brezgin S., Babin Y., Vasilyeva I., Glebe D., Kostyushev D., Chulanov V. (2022). CRISPR-Cas systems for diagnosing infectious diseases. Methods.

[12] Katzmeier F., Aufinger L., Dupin A., Quintero J., Lenz M., Bauer L.G., Klumpe S., Sherpa D., Dürr B., Honemann M. (2019). A low-cost fluorescence reader for in vitro transcription and nucleic acid detection with Cas13a. PLoS ONE.

[13] Batista A.C., Pacheco L.G. (2018). Detecting pathogens with Zinc-Finger, TALE and CRISPR- based programmable nucleic acid binding proteins. J. Microbiol. Methods.

[14] Yuan M., Ding R., Chen S., Duan G. (2021). Advances in Field Detection Based on CRISPR/Cas System. ACS Synth. Biol..

[15] Bhalla N., Jolly P., Formisano N., Estrela P. (2016). Introduction to biosensors. Essays Biochem..

[16] Kim J., Campbell A.S., de Ávila B.E.-F., Wang J. (2019). Wearable biosensors for healthcare monitoring. Nat. Biotechnol..

[17] Chen M., Luo D. (2020). A CRISPR Path to Cutting-Edge Materials. N. Engl. J. Med..

[18] Kumar S., Tripathy S., Jyoti A., Singh S.G. (2018). Recent advances in biosensors for diagnosis and detection of sepsis: A comprehensive review. Biosens. Bioelectron..

[19] Bao M., Chen Q., Xu Z., Jensen E.C., Liu C., Waitkus J.T., Yuan X., He Q., Qin P., Du K. (2021). Challenges and Opportunities for Clustered Regularly Interspaced Short Palindromic Repeats Based Molecular Biosensing. ACS Sens..

[20] Bonini A., Poma N., Vivaldi F., Biagini D., Bottai D., Tavanti A., Di Francesco F. (2021). A label-free impedance biosensing assay based on CRISPR/Cas12a collateral activity for bacterial DNA detection. J. Pharm. Biomed. Anal..

[21] Dai Y., Wu Y., Liu G., Gooding J.J. (2020). CRISPR Mediated Biosensing Toward Understanding Cellular Biology and Point-of-Care Diagnosis. Angew. Chem. Int. Ed..

[22] Cho S., Shin J., Cho B.-K. (2018). Applications of CRISPR/Cas System to Bacterial Metabolic Engineering. Int. J. Mol. Sci..

[23] Van Der Oost J., Westra E.R., Jackson R.N., Wiedenheft B. (2014). Unravelling the structural and mechanistic basis of CRISPR–Cas systems. Nat. Rev. Microbiol..

[24] Safari F., Hatam G., Behbahani A.B., Rezaei V., Barekati-Mowahed M., Petramfar P., Khademi F. (2020). CRISPR System: A High-throughput Toolbox for Research and Treatment of Parkinson’s Disease. Cell. Mol. Neurobiol..

[25] Bruch R., Urban G.A., Dincer C. (2019). CRISPR/Cas Powered Multiplexed Biosensing. Trends Biotechnol..

[26] Gootenberg J.S., Abudayyeh O.O., Lee J.W., Essletzbichler P., Dy A.J., Joung J., Verdine V., Donghia N., Daringer N.M., Freije C.A. (2017). Nucleic acid detection with CRISPR-Cas13a/C2c2. Science.

[27] Joung J., Ladha A., Saito M., Segel M., Bruneau R., Huang M.W., Kim N.-G., Yu X., Li J., Walker B.D. (2020). Point-of-care testing for COVID-19 using SHERLOCK diagnostics. medRxiv.

[28] Xie S., Ji Z., Suo T., Li B., Zhang X. (2021). Advancing sensing technology with CRISPR: From the detection of nucleic acids to a broad range of analytes—A review. Anal. Chim. Acta.

[29] Kim S., Ji S., Koh H.R. (2021). CRISPR as a Diagnostic Tool. Biomolecules.

[30] Zhang D., Yan Y., Que H., Yang T., Cheng X., Ding S., Zhang X., Cheng W. (2020). CRISPR/Cas12a-Mediated Interfacial Cleaving of Hairpin DNA Reporter for Electrochemical Nucleic Acid Sensing. ACS Sens..

[31] Akkilic N., Geschwindner S., Höök F. (2020). Single-molecule biosensors: Recent advances and applications. Biosens. Bioelectron..

[32] Thévenot D.R., Toth K., Durst R.A., Wilson G.S. (2001). Electrochemical biosensors: Recommended definitions and classification. Biosens. Bioelectron..

[33] Li Y., Li S., Wang J., Liu G. (2019). CRISPR/Cas Systems towards Next-Generation Biosensing. Trends Biotechnol..

[34] Dai Y., Somoza R., Wang L., Welter J.F., Li Y., Caplan A., Liu C.C. (2019). Exploring the Trans-Cleavage Activity of CRISPR-Cas12a (cpf1) for the Development of a Universal Electrochemical Biosensor. Angew. Chem. Int. Ed..

[35] Kadam U.S., Hong J.C. (2022). Advances in aptameric biosensors designed to detect toxic contaminants from food, water, human fluids, and the environment. Trends Environ. Anal. Chem..

[36] Gootenberg J.S., Abudayyeh O.O., Kellner M.J., Joung J., Collins J.J., Zhang F. (2018). Multiplexed and portable nucleic acid detection platform with Cas13, Cas12a, and Csm6. Science.

[37] Murugan K., Babu K., Sundaresan R., Rajan R., Sashital D.G. (2017). The Revolution Continues: Newly Discovered Systems Expand the CRISPR-Cas Toolkit. Mol. Cell.

[38] Wu H., Chen X., Zhang M., Wang X., Chen Y., Qian C., Wu J., Xu J. (2021). Versatile detection with CRISPR/Cas system from applications to challenges. TrAC Trends Anal. Chem..

[39] Swetha P.D.P., Sonia J., Sapna K., Prasad K.S. (2021). Towards CRISPR powered electrochemical sensing for smart diagnostics. Curr. Opin. Electrochem..

[40] Zhang Y., Wu Y., Wu Y., Chang Y., Liu M. (2021). CRISPR-Cas systems: From gene scissors to programmable biosensors. TrAC Trends Anal. Chem..

[41] Wang J. (2006). Electrochemical biosensors: Towards point-of-care cancer diagnostics. Biosens. Bioelectron..

[42] Lazaro A., Boada M., Villarino R., Girbau D. (2019). Color Measurement and Analysis of Fruit with a Battery-Less NFC Sensor. Sensors.

[43] Moon J., Kwon H.-J., Yong D., Lee I.-C., Kim H., Kang H., Lim E.-K., Lee K.-S., Jung J., Park H.G. (2020). Colorimetric Detection of SARS-CoV-2 and Drug-Resistant pH1N1 Using CRISPR/dCas9. ACS Sens..

[44] Cao Y., Wu J., Pang B., Zhang H., Le X.C. (2021). CRISPR/Cas12a-mediated gold nanoparticle aggregation for colorimetric detection of SARS-CoV-2. Chem. Commun..

[45] Shi Y., Fu X., Yin Y., Peng F., Yin X., Ke G., Zhang X. (2021). CRISPR-Cas12a System for Biosensing and Gene Regulation. Chem. Asian J..

[46] Cheng M., Xiong E., Tian T., Zhu D., Ju H.-Q., Zhou X. (2021). A CRISPR-driven colorimetric code platform for highly accurate telomerase activity assay. Biosens. Bioelectron..

[47] Jiang Y., Hu M., Liu A.-A., Lin Y., Liu L., Yu B., Zhou X., Pang D.-W. (2021). Detection of SARS-CoV-2 by CRISPR/Cas12a-Enhanced Colorimetry. ACS Sens..

[48] Nalefski E.A., Patel N., Leung P.J., Islam Z., Kooistra R.M., Parikh I., Marion E., Knott G.J., Doudna J.A., Le Ny A.-L.M. (2021). Kinetic analysis of Cas12a and Cas13a RNA-Guided nucleases for development of improved CRISPR-Based diagnostics. iScience.

[49] Chen J.S., Ma E., Harrington L.B., Da Costa M., Tian X., Palefsky J.M., Doudna J.A. (2018). CRISPR-Cas12a target binding unleashes indiscriminate single-stranded DNase activity. Science.

[50] Zetsche B., Gootenberg J.S., Abudayyeh O.O., Slaymaker I.M., Makarova K.S., Essletzbichler P., Volz S.E., Joung J., van der Oost J., Regev A. (2015). Cpf1 Is a Single RNA-Guided Endonuclease of a Class 2 CRISPR-Cas System. Cell.

[51] Mustafa M.I., Makhawi A.M. (2021). SHERLOCK and DETECTR: CRISPR-Cas Systems as Potential Rapid Diagnostic Tools for Emerging Infectious Diseases. J. Clin. Microbiol..

[52] Kellner M.J., Koob J.G., Gootenberg J.S., Abudayyeh O.O., Zhang F. (2019). SHERLOCK: Nucleic acid detection with CRISPR nucleases. Nat. Protoc..

[53] Wang C., Horby P.W., Hayden F.G., Gao G.F. (2020). A novel coronavirus outbreak of global health concern. Lancet.

[54] Yin L., Man S., Ye S., Liu G., Ma L. (2021). CRISPR-Cas based virus detection: Recent advances and perspectives. Biosens. Bioelectron..

[55] Zhu N., Zhang D., Wang W., Li X., Yang B., Song J., Zhao X., Huang B., Shi W., Lu R. (2020). A Novel Coronavirus from Patients with Pneumonia in China, 2019. N. Engl. J. Med..

[56] Bhattacharyya R.P., Thakku S.G., Hung D.T. (2018). Harnessing CRISPR Effectors for Infectious Disease Diagnostics. ACS Infect. Dis..

[57] Quan J., Langelier C., Kuchta A., Batson J., Teyssier N., Lyden A., Caldera S., McGeever A., Dimitrov B., King R. (2019). FLASH: A next-generation CRISPR diagnostic for multiplexed detection of antimicrobial resistance sequences. Nucleic Acids Res..

[58] Ma L., Peng L., Yin L., Liu G., Man S. (2021). CRISPR-Cas12a-Powered Dual-Mode Biosensor for Ultrasensitive and Cross-validating Detection of Pathogenic Bacteria. ACS Sens..

[59] Li Z., Wei J., Di D., Wang X., Li C., Li B., Qiu Y., Liu K., Gu F., Tong M. (2020). Rapid and accurate detection of African swine fever virus by DNA endonuclease-targeted CRISPR trans reporter assay. Acta Biochim. Biophys. Sin..

[60] Escalona-Noguero C., López-Valls M., Sot B. (2021). CRISPR/Cas technology as a promising weapon to combat viral infections. Bioessays.

[61] Nishibuchi M. (2012). Food poisoning—Importance of international perspective. Nihon Rinsho. Jpn. J. Clin. Med..

[62] Rosegrant M.W., Cline S.A. (2003). Global Food Security: Challenges and Policies. Science.

[63] Liu X., Bu S., Feng J., Wei H., Wang Z., Li X., Zhou H., He X., Wan J. (2021). Electrochemical biosensor for detecting pathogenic bacteria based on a hybridization chain reaction and CRISPR-Cas12a. Anal. Bioanal. Chem..

[64] De Freitas C.G., Santana P., da Silva P.H.C., Gonçalves V.S.P., Barros M.D.A.F., Torres F.A.G., Murata L.S., Perecmanis S. (2010). PCR multiplex for detection of Salmonella Enteritidis, Typhi and Typhimurium and occurrence in poultry meat. Int. J. Food Microbiol..

[65] Fu X., Sun J., Ye Y., Zhang Y., Sun X. (2022). A rapid and ultrasensitive dual detection platform based on Cas12a for simultaneous detection of virulence and resistance genes of drug-resistant Salmonella. Biosens. Bioelectron..

[66] Sun X., Wang Y., Zhang L., Liu S., Zhang M., Wang J., Ning B., Peng Y., He J., Hu Y. (2020). CRISPR-Cas9 Triggered Two-Step Isothermal Amplification Method for *E. coli* O157:H7 Detection Based on a Metal–Organic Framework Platform. Anal. Chem..

[67] Zhou J., Yin L., Dong Y., Peng L., Liu G., Man S., Ma L. (2020). CRISPR-Cas13a based bacterial detection platform: Sensing pathogen Staphylococcus aureus in food samples. Anal. Chim. Acta.

[68] Yan M.-Y., Li S.-S., Ding X.-Y., Guo X.-P., Jin Q., Sun Y.-C. (2020). A CRISPR-Assisted Nonhomologous End-Joining Strategy for Efficient Genome Editing in Mycobacterium tuberculosis. mBio.

[69] Wei J., Lu N., Li Z., Wu X., Jiang T., Xu L., Yang C., Guo S. (2019). The *Mycobacterium tuberculosis* CRISPR-Associated Cas1 Involves Persistence and Tolerance to Anti-Tubercular Drugs. BioMed Res. Int..

[70] Grüschow S., Athukoralage J., Graham S., Hoogeboom T., White M.F. (2019). Cyclic oligoadenylate signalling mediates *Mycobacterium tuberculosis* CRISPR defence. Nucleic Acids Res..

[71] Ai J.-W., Zhou X., Xu T., Yang M., Chen Y., He G.-Q., Pan N., Cai Y., Li Y., Wang X. (2019). CRISPR-based rapid and ultra-sensitive diagnostic test for *Mycobacterium tuberculosis*. Emerg. Microbes Infect..

[72] Bertelli C., Cissé O.H., Rusconi B., Kebbi-Beghdadi C., Croxatto A., Goesmann A., Collyn F., Greub G. (2016). CRISPR System Acquisition and Evolution of an Obligate Intracellular *Chlamydia*-Related Bacterium. Genome Biol. Evol..

[73] Ouellette S.P. (2018). Feasibility of a Conditional Knockout System for Chlamydia Based on CRISPR Interference. Front. Cell. Infect. Microbiol..

[74] Ouellette S.P., Blay E.A., Hatch N.D., Fisher-Marvin L.A. (2021). CRISPR Interference To Inducibly Repress Gene Expression in Chlamydia trachomatis. Infect. Immun..

[75] Bangpanwimon K., Sottisuporn J., Mittraparp-Arthorn P., Ueaphatthanaphanich W., Rattanasupar A., Pourcel C., Vuddhakul V. (2017). CRISPR-like sequences in *Helicobacter pylori* and application in genotyping. Gut Pathog..

[76] Waskito L.A., Salama N.R., Yamaoka Y. (2018). Pathogenesis of *Helicobacter pylori* infection. Helicobacter.

[77] Kao C.-Y., Sheu B.-S., Wu J.-J. (2016). *Helicobacter pylori* infection: An overview of bacterial virulence factors and pathogenesis. Biomed. J..

[78] García-Zea J.A., De La Herrán R., Rodríguez F.R., Navajas-Pérez R., Rejón C.R. (2019). Detection and variability analyses of CRISPR-like loci in the *H. pylori* genome. PeerJ.

[79] Zawilak-Pawlik A., Zakrzewska-Czerwińska J. (2017). Recent Advances in *Helicobacter pylori* Replication: Possible Implications in Adaptation to a Pathogenic Lifestyle and Perspectives for Drug Design. Curr. Top Microbiol. Immunol..

[80] Wang Y.-K., Kuo F.-C., Liu C.-J., Wu M.-C., Shih H.-Y., Wang S.S., Wu J.-Y., Kuo C.-H., Huang Y.-K., Wu D.-C. (2015). Diagnosis of *Helicobacter pylori* infection: Current options and developments. World J. Gastroenterol..

[81] Khalilpour A., Kazemzadeh-Narbat M., Tamayol A., Oklu R., Khademhosseini A. (2016). Biomarkers and diagnostic tools for detection of *Helicobacter pylori*. Appl. Microbiol. Biotechnol..

[82] Yee J.K. (2016). *Helicobacter pylori* colonization of the oral cavity: A milestone discovery. World J. Gastroenterol..

[83] Kawai S., Arai K., Lin Y., Nishiyama T., Sasakabe T., Wang C., Miwa H., Kikuchi S. (2019). Comparison of the detection of *Helicobacter pylori* infection by commercially available serological testing kits and the 13C-urea breath test. J. Infect. Chemother..

[84] Kosunen T., Seppäla K., Sarna S., Sipponen P. (1992). Diagnostic value of decreasing IgG, IgA, and IgM antibody titres after eradication of *Helicobacter pylori*. Lancet.

[85] Beer-Davidson G., Hindiyeh M., Muhsen K. (2018). Detection of *Helicobacter pylori* in stool samples of young children using real-time polymerase chain reaction. Helicobacter.

[86] Mentis A., Lehours P., Mégraud F. (2015). Epidemiology and Diagnosis of *Helicobacter pylori* infection. Helicobacter.

[87] Vidic J., Manzano M. (2021). Electrochemical biosensors for rapid pathogen detection. Curr. Opin. Electrochem..

[88] Saxena K., Chauhan N., Jain U. (2021). Advances in diagnosis of *Helicobacter pylori* through biosensors: Point of care devices. Anal. Biochem..

[89] Liu Z., Su X. (2017). A novel fluorescent DNA sensor for ultrasensitive detection of *Helicobacter pylori*. Biosens. Bioelectron..

[90] Ly S.Y., Yoo H.-S., Choa S.H. (2011). Diagnosis of *Helicobacter pylori* bacterial infections using a voltammetric biosensor. J. Microbiol. Methods.

[91] Ali M.M., Wolfe M., Tram K., Gu J., Filipe C.D.M., Li Y., Brennan J.D. (2019). A DNAzyme-Based Colorimetric Paper Sensor for *Helicobacter pylori*. Angew. Chem. Int. Ed..

[92] Del Pozo M.V., Alonso C., Pariente F., Lorenzo E. (2005). DNA Biosensor for Detection of *Helicobacter pylori* Using Phen-dione as the Electrochemically Active Ligand in Osmium Complexes. Anal. Chem..

[93] Hajihosseini S., Nasirizadeh N., Hejazi M.S., Yaghmaei P. (2016). An electrochemical DNA biosensor based on Oracet Blue as a label for detection of *Helicobacter pylori*. Int. J. Biol. Macromol..

[94] Dindar B., Karakuş E., Abasıyanık F. (2011). New Urea Biosensor Based on Urease Enzyme Obtained from *Helycobacter pylori*. Appl. Biochem. Biotechnol..

[95] Vangah S.J., Katalani C., Boone H.A., Hajizade A., Sijercic A., Ahmadian G. (2020). CRISPR-Based Diagnosis of Infectious and Noninfectious Diseases. Biol. Proced. Online.

[96] Qiu E., Jin S., Xiao Z., Chen Q., Wang Q., Liu H., Xie C., Chen C., Li Z., Han S. (2021). CRISPR-based detection of *Helicobacter pylori* in stool samples. Helicobacter.

[97] Gregorio G.V., Mieli-Vergani G., Mowat A.P. (1994). Viral hepatitis. Arch. Dis. Child..

[98] Kumar H., Kamar N., Kumar D. (2019). Hepatitis E: Current Status in India and Other Asian Countries. J. Pure Appl. Microbiol..

[99] Pardee M. (2019). Diagnosis and Management of Hepatitis B and C. Nurs. Clin. N. Am..

[100] Shariati M., Sadeghi M. (2020). Ultrasensitive DNA biosensor for hepatitis B virus detection based on tin-doped WO_3_/In_2_O_3_ heterojunction nanowire photoelectrode under laser amplification. Anal. Bioanal. Chem..

[101] Yao C.-Y. (2014). Biosensors for hepatitis B virus detection. World J. Gastroenterol..

[102] Manzano M., Viezzi S., Mazerat S., Marks R.S., Vidic J. (2018). Rapid and label-free electrochemical DNA biosensor for detecting hepatitis A virus. Biosens. Bioelectron..

[103] Riedel T., Surman F., Hageneder S., Pop-Georgievski O., Noehammer C., Hofner M., Brynda E., Rodriguez-Emmenegger C., Dostálek J. (2016). Hepatitis B plasmonic biosensor for the analysis of clinical serum samples. Biosens. Bioelectron..

[104] Timurdogan E., Alaca B.E., Kavakli I.H., Urey H. (2011). MEMS biosensor for detection of Hepatitis A and C viruses in serum. Biosens. Bioelectron..

[105] Chowdhury A.D., Takemura K., Li T.-C., Suzuki T., Park E.Y. (2019). Electrical pulse-induced electrochemical biosensor for hepatitis E virus detection. Nat. Commun..

[106] Tang D., Tang J., Su B., Ren J., Chen G. (2010). Simultaneous determination of five-type hepatitis virus antigens in 5min using an integrated automatic electrochemical immunosensor array. Biosens. Bioelectron..

[107] Chen X., Tan Y., Wang S., Wu X., Liu R., Yang X., Wang Y., Tai J., Li S. (2021). A CRISPR-Cas12b–Based Platform for Ultrasensitive, Rapid, and Highly Specific Detection of Hepatitis B Virus Genotypes B and C in Clinical Application. Front. Bioeng. Biotechnol..

[108] Gong S., Zhang S., Wang X., Li J., Pan W., Li N., Tang B. (2021). Strand Displacement Amplification Assisted CRISPR-Cas12a Strategy for Colorimetric Analysis of Viral Nucleic Acid. Anal. Chem..

[109] Wang S., Li H., Kou Z., Ren F., Jin Y., Yang L., Dong X., Yang M., Zhao J., Dong N. (2021). Highly sensitive and specific detection of hepatitis B virus DNA and drug resistance mutations utilizing the PCR-based CRISPR-Cas13a system. Clin. Microbiol. Infect..

[110] Choi J.-H., Shin M., Yang L., Conley B., Yoon J., Lee S.-N., Lee K.-B., Choi J.-W. (2021). Clustered Regularly Interspaced Short Palindromic Repeats-Mediated Amplification-Free Detection of Viral DNAs Using Surface-Enhanced Raman Spectroscopy-Active Nanoarray. ACS Nano.

[111] Ding R., Long J., Yuan M., Zheng X., Shen Y., Jin Y., Yang H., Li H., Chen S., Duan G. (2021). CRISPR/Cas12-Based Ultra-Sensitive and Specific Point-of-Care Detection of HBV. Int. J. Mol. Sci..

[112] Herrera-Carrillo E., Gao Z., Berkhout B. (2020). CRISPR therapy towards an HIV cure. Brief. Funct. Genom..

[113] Saayman S., Ali S., Morris K., Weinberg M. (2015). The therapeutic application of CRISPR/Cas9 technologies for HIV. Expert Opin. Biol. Ther..

[114] Yin L., Zhao F., Sun H., Wang Z., Huang Y., Zhu W., Xu F., Mei S., Liu X., Zhang D. (2020). CRISPR-Cas13a Inhibits HIV-1 Infection. Mol. Ther. Nucleic Acids.

[115] Li H., Wang S., Dong X., Li Q., Li M., Li J., Guo Y., Jin X., Zhou Y., Song H. (2020). CRISPR-Cas13a Cleavage of Dengue Virus NS3 Gene Efficiently Inhibits Viral Replication. Mol. Ther. Nucleic Acids.

[116] Kistler K.E., Vosshall L.B., Matthews B.J. (2015). Genome Engineering with CRISPR-Cas9 in the Mosquito Aedes aegypti. Cell Rep..

[117] Carlin A.F., Shresta S. (2019). Genome-wide approaches to unravelling host–virus interactions in Dengue and Zika infections. Curr. Opin. Virol..

[118] Gong J., Zhang G., Wang W., Liang L., Li Q., Liu M., Xue L., Tang G. (2021). A simple and rapid diagnostic method for 13 types of high-risk human papillomavirus (HR-HPV) detection using CRISPR-Cas12a technology. Sci. Rep..

[119] Maver P., Poljak M. (2020). Primary HPV-based cervical cancer screening in Europe: Implementation status, challenges, and future plans. Clin. Microbiol. Infect..

[120] Li Z., Ding X., Yin K., Xu Z., Cooper K., Liu C. (2021). Electric field-enhanced electrochemical CRISPR biosensor for DNA detection. Biosens. Bioelectron..

[121] Tsou J.-H., Leng Q., Jiang F. (2019). A CRISPR Test for Detection of Circulating Nuclei Acids. Transl. Oncol..

[122] Wang L., Shen X., Wang T., Chen P., Qi N., Yin B.-C., Ye B.-C. (2020). A lateral flow strip combined with Cas9 nickase-triggered amplification reaction for dual food-borne pathogen detection. Biosens. Bioelectron..

[123] Zhang Y., Qian L., Wei W., Wang Y., Wang B., Lin P., Liu W., Yixuan Y., Li X., Liu D. (2017). Paired Design of dCas9 as a Systematic Platform for the Detection of Featured Nucleic Acid Sequences in Pathogenic Strains. ACS Synth. Biol..

[124] Liu H., Wang J., Hu X., Tang X., Zhang C. (2022). A rapid and high-throughput *Helicobacter pylori* RPA-CRISPR/Cas12a-based nucleic acid detection system. Clin. Chim. Acta.

[125] Kachwala M.J., Smith C.W., Nandu N., Yigit M.V. (2021). Reprogrammable Gel Electrophoresis Detection Assay Using CRISPR-Cas12a and Hybridization Chain Reaction. Anal. Chem..

[126] Zhou R., Li Y., Dong T., Tang Y., Li F. (2020). A sequence-specific plasmonic loop-mediated isothermal amplification assay with orthogonal color readouts enabled by CRISPR Cas12a. Chem. Commun..

[127] Shao N., Han X., Song Y., Zhang P., Qin L. (2019). CRISPR-Cas12a Coupled with Platinum Nanoreporter for Visual Quantification of SNVs on a Volumetric Bar-Chart Chip. Anal. Chem..

[128] Lee H., Choi J., Jeong E., Baek S., Kim H.C., Chae J.-H., Koh Y., Seo S.W., Kim J.-S., Kim S.J. (2018). dCas9-mediated Nanoelectrokinetic Direct Detection of Target Gene for Liquid Biopsy. Nano Lett..

[129] Nouri R., Jiang Y., Lian X.L., Guan W. (2020). Sequence-Specific Recognition of HIV-1 DNA with Solid-State CRISPR-Cas12a-Assisted Nanopores (SCAN). ACS Sens..

[130] Pardee K., Green A.A., Takahashi M.K., Braff D., Lambert G., Lee J.W., Ferrante T., Ma D., Donghia N., Fan M. (2016). Rapid, Low-Cost Detection of Zika Virus Using Programmable Biomolecular Components. Cell.

[131] Ackerman C.M., Myhrvold C., Thakku S.G., Freije C.A., Metsky H.C., Yang D.K., Ye S.H., Boehm C.K., Kosoko-Thoroddsen T.-S.F., Kehe J. (2020). Massively multiplexed nucleic acid detection with Cas13. Nature.

[132] Myhrvold C., Freije C.A., Gootenberg J.S., Abudayyeh O.O., Metsky H.C., Durbin A.F., Kellner M.J., Tan A.L., Paul L.M., Parham L.A. (2018). Field-deployable viral diagnostics using CRISPR-Cas13. Science.

[133] Zhang B., Wang Q., Xu X., Xia Q., Long F., Li W., Shui Y., Xia X., Wang J. (2018). Detection of target DNA with a novel Cas9/sgRNAs-associated reverse PCR (CARP) technique. Anal. Bioanal. Chem..

[134] Teng F., Guo L., Cui T., Wang X.-G., Xu K., Gao Q., Zhou Q., Li W. (2019). CDetection: CRISPR-Cas12b-based DNA detection with sub-attomolar sensitivity and single-base specificity. Genome Biol..

[135] Zhang B., Xia Q., Wang Q., Xia X., Wang J. (2018). Detecting and typing target DNA with a novel CRISPR-typing PCR (ctPCR) technique. Anal. Biochem..

[136] Yin K., Ding X., Li Z., Zhao H., Cooper K., Liu C. (2020). Dynamic Aqueous Multiphase Reaction System for One-Pot CRISPR-Cas12a-Based Ultrasensitive and Quantitative Molecular Diagnosis. Anal. Chem..

[137] Rai P., Kumar B.K., Deekshit V.K., Karunasagar I., Karunasagar I. (2021). Detection technologies and recent developments in the diagnosis of COVID-19 infection. Appl. Microbiol. Biotechnol..

[138] Yesudhas D., Srivastava A., Gromiha M.M. (2021). COVID-19 outbreak: History, mechanism, transmission, structural studies and therapeutics. Infection.

[139] Corman V.M., Landt O., Kaiser M., Molenkamp R., Meijer A., Chu D.K.W., Bleicker T., Brünink S., Schneider J., Schmidt M.L. (2020). Detection of 2019 novel coronavirus (2019-nCoV) by real-time RT-PCR. Eurosurveillance.

[140] Behera B.C., Mishra R.R., Thatoi H. (2021). Recent biotechnological tools for diagnosis of corona virus disease: A review. Biotechnol. Prog..

[141] McCormick-Baw C., Morgan K., Gaffney D., Cazares Y., Jaworski K., Byrd A., Molberg K., Cavuoti D. (2020). Saliva as an Alternate Specimen Source for Detection of SARS-CoV-2 in Symptomatic Patients Using Cepheid Xpert Xpress SARS-CoV-2. J. Clin. Microbiol..

[142] Ali N., Rampazzo R., Costa A.D.T., Krieger M.A. (2017). Current Nucleic Acid Extraction Methods and Their Implications to Point-of-Care Diagnostics. BioMed Res. Int..

[143] Ravi N., Cortade D.L., Ng E., Wang S.X. (2020). Diagnostics for SARS-CoV-2 detection: A comprehensive review of the FDA-EUA COVID-19 testing landscape. Biosens. Bioelectron..

[144] Nouri R., Tang Z., Dong M., Liu T., Kshirsagar A., Guan W. (2021). CRISPR-based detection of SARS-CoV-2: A review from sample to result. Biosens. Bioelectron..

[145] Xiong Y., Zhang J., Yang Z., Mou Q., Ma Y., Xiong Y., Lu Y. (2020). Functional DNA Regulated CRISPR-Cas12a Sensors for Point-of-Care Diagnostics of Non-Nucleic-Acid Targets. J. Am. Chem. Soc..

[146] Lucia C., Federico P.B., Alejandra G.C. (2020). An ultrasensitive, rapid, and portable coronavirus SARS-CoV-2 sequence detection method based on CRISPR-Cas12. bioRxiv.

[147] Ding X., Bin P., Wu W., Chang Y., Zhu G. (2020). Tryptophan Metabolism, Regulatory T Cells, and Inflammatory Bowel Disease: A Mini Review. Mediat. Inflamm..

[148] Broughton J.P., Deng X., Yu G., Fasching C.L., Servellita V., Singh J., Miao X., Streithorst J.A., Granados A., Sotomayor-Gonzalez A. (2020). CRISPR–Cas12-based detection of SARS-CoV-2. Nat. Biotechnol..

[149] Ali Z., Aman R., Mahas A., Rao G.S., Tehseen M., Marsic T., Salunke R., Subudhi A.K., Hala S.M., Hamdan S.M. (2020). iSCAN: An RT-LAMP-coupled CRISPR-Cas12 module for rapid, sensitive detection of SARS-CoV-2. Virus Res..

[150] Guo L., Liu W., Li Z., Li L. An adaptive sliding mode control strategy for the heading control of autonomous underwater vehicles. Proceedings of the Global Oceans 2020: Singapore—U.S. Gulf Coast.

[151] Rauch J.N., Valois E., Solley S.C., Braig F., Lach R.S., Audouard M., Ponce-Rojas J.C., Costello M.S., Baxter N.J., Kosik K.S. (2021). A Scalable, Easy-to-Deploy Protocol for Cas13-Based Detection of SARS-CoV-2 Genetic Material. J. Clin. Microbiol..

[152] Hou T., Zeng W., Yang M., Chen W., Ren L., Ai J., Wu J., Liao Y., Gou X., Li Y. (2020). Development and evaluation of a rapid CRISPR-based diagnostic for COVID-19. PLoS Pathog..

[153] Patchsung M., Jantarug K., Pattama A., Aphicho K., Suraritdechachai S., Meesawat P., Sappakhaw K., Leelahakorn N., Ruenkam T., Wongsatit T. (2020). Clinical validation of a Cas13-based assay for the detection of SARS-CoV-2 RNA. Nat. Biomed. Eng..

[154] Hayden C., Metsky C.A., Freije T., Solveig F., Kosoko-Thoroddsen P.C., Sabeti C. (2020). Myhrvold CRISPR-based surveillance for COVID-19 using genomically-comprehensive machine learning design. bioRxiv.

[155] Arizti-Sanz J., Freije C.A., Stanton A.C., Boehm C.K., Petros B.A., Siddiqui S., Shaw B.M., Adams G., Kosoko-Thoroddsen T.F., Kemball M.E. (2020). Integrated sample inactivation, amplification, and Cas13-based detection of SARS-CoV-2. bioRxiv.

[156] Aman R., Mahas A., Mahfouz M. (2020). Nucleic Acid Detection Using CRISPR/Cas Biosensing Technologies. ACS Synth. Biol..

[157] Diego J.G.-B., Fernández-Soto P., Muro A. (2022). The Future of Point-of-Care Nucleic Acid Amplification Diagnostics after COVID-19: Time to Walk the Walk. Int. J. Mol. Sci..

[158] Kaminski M.M., Abudayyeh O.O., Gootenberg J.S., Zhang F., Collins J.J. (2021). CRISPR-based diagnostics. Nat. Biomed. Eng..

[159] Qi Y., Li K., Li Y., Guo D., Xu J., Li Y., Gong W. (2022). CRISPR-Based Diagnostics: A Potential Tool to Address the Diagnostic Challenges of Tuberculosis. Pathogens.

